# Dual UMIs and Dual Barcodes With Minimal PCR Amplification Removes Artifacts and Acquires Accurate Antibody Repertoire

**DOI:** 10.3389/fimmu.2021.778298

**Published:** 2021-12-22

**Authors:** Qilong Wang, Huikun Zeng, Yan Zhu, Minhui Wang, Yanfang Zhang, Xiujia Yang, Haipei Tang, Hongliang Li, Yuan Chen, Cuiyu Ma, Chunhong Lan, Bin Liu, Wei Yang, Xueqing Yu, Zhenhai Zhang

**Affiliations:** ^1^ Center for Precision Medicine, Guangdong Provincial People’s Hospital, Guangdong Academy of Medical Sciences, Guangzhou, China; ^2^ Guangdong-Hong Kong Joint Laboratory on Immunological and Genetic Kidney Diseases, Guangdong Provincial People’s Hospital, Guangdong Academy of Medical Sciences, Guangzhou, China; ^3^ State Key Laboratory of Organ Failure Research, National Clinical Research Center for Kidney Disease, Division of Nephrology, Nanfang Hospital, Southern Medical University, Guangzhou, China; ^4^ Department of Bioinformatics, School of Basic Medical Sciences, Southern Medical University, Guangzhou, China; ^5^ School of Computer Science and Technology, Beijing Institute of Technology, Beijing, China; ^6^ Department of Pathology, School of Basic Medical Sciences, Southern Medical University, Guangzhou, China; ^7^ Division of Nephrology, Guangdong Provincial People’s Hospital, Guangdong Academy of Medical Sciences, Guangzhou, China; ^8^ Key Laboratory of Mental Health of the Ministry of Education, Guangdong-Hong Kong-Macao Greater Bay Area Center for Brain Science and Brain-Inspired Intelligence, Southern Medical University, Guangzhou, China

**Keywords:** antibody repertoire, rep-seq, chimera, sequencing error, unique molecular identifier (UMI)

## Abstract

Antibody repertoire sequencing (Rep-seq) has been widely used to reveal repertoire dynamics and to interrogate antibodies of interest at single nucleotide-level resolution. However, polymerase chain reaction (PCR) amplification introduces extensive artifacts including chimeras and nucleotide errors, leading to false discovery of antibodies and incorrect assessment of somatic hypermutations (SHMs) which subsequently mislead downstream investigations. Here, a novel approach named DUMPArts, which improves the accuracy of antibody repertoires by labeling each sample with dual barcodes and each molecule with dual unique molecular identifiers (UMIs) *via* minimal PCR amplification to remove artifacts, is developed. Tested by ultra-deep Rep-seq data, DUMPArts removed inter-sample chimeras, which cause artifactual shared clones and constitute approximately 15% of reads in the library, as well as intra-sample chimeras with erroneous SHMs and constituting approximately 20% of the reads, and corrected base errors and amplification biases by consensus building. The removal of these artifacts will provide an accurate assessment of antibody repertoires and benefit related studies, especially mAb discovery and antibody-guided vaccine design.

## Introduction

Antibodies (Abs), also known as immunoglobulins (Igs), are the most important component of humoral immunity. An antibody can neutralize a pathogen by recognizing a unique component (antigen) of the pathogen *via* its fragment antigen-binding (Fab) variable region. The entire set of antibodies within an individual or tissue constitutes a tremendously diverse antibody repertoire. During B cell development, somatic recombination of variable (V), diversity (D, for heavy chain only) and joining (J) gene segments, non-templated (N) or palindromic (P) addition or subtraction of nucleotides at the junctions, and class switch recombination (CSR) and somatic hypermutation (SHM) upon activation all contribute to the diversity of the antibody repertoire (1, 2). This diversity enables B cells to recognize and neutralize a wide range of antigens, particularly invading pathogens and autoantigens accumulated in the body (3, 4). Accurately characterizing and quantifying the antibody repertoire are vital to discovering antibodies that recognize specific antigens of interest, including virus-neutralizing antibodies (5–7) and therapeutic antibodies (8, 9), guiding the development of vaccines (10), detecting B-cell malignancies with high sensitivity (11), and monitoring immune status (9).

Recent advances in high-throughput sequencing (HTS) of antibody repertoire (Rep-seq or AIRR-seq, a term coined by the AIRR Community) have enabled researchers to decipher the antibody repertoire on an unprecedented scale (12, 13). Several Rep-seq strategies, including bulk Rep-seq, single-cell Rep-seq [including LIBRA-seq (linking B cell receptor to antigen specificity through sequencing) (7) and OE RT-PCR (overlap extension reverse transcription polymerase chain reaction) (14)], have been developed for different applications. Although native pair information is lost, bulk Rep-seq remains the most widely used approach due to its low-cost, ease of application, potential for high throughput, and its ability to obtain full-length variable region sequences (1, 15, 16). One of the major challenges of bulk Rep-seq is reducing artifactual sequences introduced by PCR amplification and HTS. Upon amplification of a mixture of similar sequences, a considerable number of chimeras, accounting for over 30% of all sequences, were introduced due to template switching and PCR-mediated recombination (17–24), which substantially impacts our understanding of the antibody repertoire, including analyses of V gene assignment and SHM frequency (1), evaluations of clonal expansion and diversity, discovery of antigen-specific mAbs, and elucidation of the antibody maturation pathway. Therefore, the quantitative assessment of chimeras in Rep-seq data deserves serious attention, and their elimination is of great importance for extracting the most pertinent biological information from an antibody repertoire.

Chimeras in Rep-seq applications can be classified into three categories: inter-library chimeras, inter-sample (same library) chimeras, and intra-sample chimeras. Several strategies have been suggested to remove inter-library and inter-sample chimeras (25–29). For example, unique dual indices offered by Illumina can minimize inter-library chimeras (induced by index hopping) by labeling each library with unique paired indices and data splitting (25, 27). Similarly, dual indices (barcodes) can be applied to remove inter-sample chimeras (26, 28, 29). However, removing intra-sample chimeras generated during PCR amplification in Rep-seq is extremely challenging. Previous studies of chimeras with very few sequences used sequence alignment to reveal chimera formation (24). This method is not applicable to Rep-seq data because the V, D, and J genes that give rise to antibody diversity are highly similar to one other (1, 2, 30). Therefore, there is a tremendous unmet need for a strategy that can quantify and remove intra-sample chimeras.

In addition to chimeras, another challenge of Rep-seq is correcting base errors and amplification biases introduced by PCR amplification and HTS, which is fundamental for characterizing SHMs, quantifying rare antibodies, and understanding antibody repertoires. It’s reported that the substitution errors for amplicon sequencing have been greatly corrected by using quality score combined with Hamming graph and read overlapping (31). Moreover, amplification biases have been largely addressed by the introduction of unique molecular identifiers (UMIs), the random-tandem sequences with huge diversity, during reverse transcription (RT), thus subsequent PCR amplification of each cDNA molecule can be quantified and corrected by grouping antibodies based on UMIs or UMI pairs and subsequent consensus sequence building (28, 29, 32–38). Besides, with the capability of tracking individual RNA molecules throughout PCR amplification and sequencing (33, 35, 39), UMIs also possess the potential for identifying and removing intra-sample chimeras. Previous methods incorporating UMIs are either single-end UMI labeling strategy (29) that could not identify intra-sample chimeras or dual UMIs labeling strategy introduced by multiple cycles of PCR amplification (36, 38) which lead to the loss of the unique labeling characteristics of UMI. Additionally, there is another dual UMI strategy which labels each antibody RNA molecule uniquely but cannot acquire the full-length of the antibody variable region (35). Hence, a strategy that fulfils the goals of labeling each molecule uniquely, acquiring full-length variable regions, and quantifying and removing intra-sample chimeras is need. In addition, although previous methods removed singletons or UMI groups with read numbers less than 3 for preliminary error correction, they mainly focused on building consensus sequences to eliminate base errors and amplification bias. And these methods, with the threshold of UMI greater than 1 or 3, cannot remove the chimeras introduced by early PCR cycles.

Here, we describe a novel experimental and computational strategy termed DUMPArts that labels each molecule with unique UMI pair using the strategy that one UMI was introduced during reverse transcription (RT) and another UMI during second-strand cDNA synthesis with only one cycle PCR extension. DUMPArts facilitates the removal of inter-sample chimeras using the barcode pair and the intra-sample chimeras using the distribution of number of reads per UMI pair (RPUP), which removes chimeras more flexibly and thoroughly, compared with previous studies that just removing singletons or UMI groups with read number less than 3. Moreover, due to the introduction of UMIs, DUMPArts corrects base errors and amplification biases by consensus sequence building, and provides, for the first time, true full-length variable regions within an antibody repertoire. Utilizing this powerful approach, we can accurately interpret the characteristics of the antibody repertoire, which is critical for antibody-guided vaccine design, antigen-specific antibody screening, detection of B-cell malignancies, and other related applications.

## Results

### Donors Within the Same Project Shared More Low-Frequency Clones With Higher SHMs

**Figure 1:**
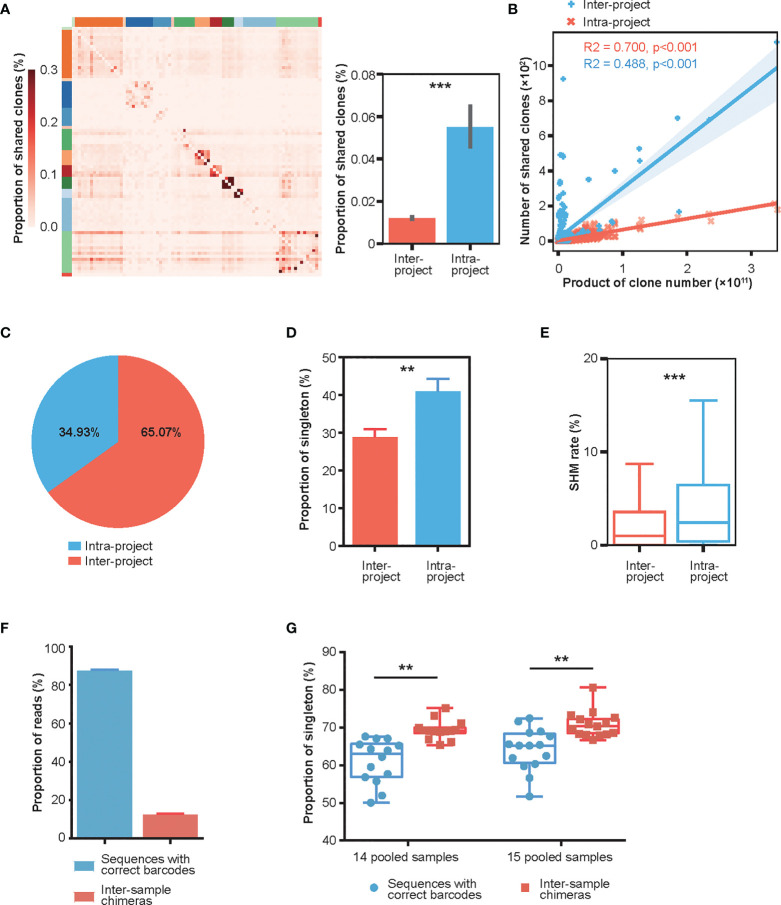
Characterization of intra- and inter-project “shared clones”. **(A)** Proportion distribution of between-donor “shared clones” in 14 published Rep-seq projects where 83 donors were grouped based on the projects (marked by different colors on the top and left) and arranged in the same order on the X and Y axes. The color represents the proportion of shared clones between the corresponding donors on the X-axis and Y-axis to the total clones in the donor at the X-axis. The right panel shows that the proportions of intra-project shared clones (n = 690) is much greater than their inter-project counterparts (n = 3996). ***P < 0.001 (unpaired t-test, mean ± s.e.m.). **(B)** Linear fitting of the number of “shared clones” as a function of the products of clone numbers of their corresponding sample pairs. **(C)** Composition of the shared clones. **(D)** Proportion of “shared clones” consisting of singletons (clones with only one read). **P < 0.01 (unpaired t-test, mean ± s.e.m.). **(E)** SHM rates of the intra-project (n = 54447) and inter-project (n = 4858) shared clones. ***P < 0.001 (unpaired t-test). **(F)** Distribution of Rep-seq reads with correct and incorrect barcode pairs after library construction using six cycles of PCR amplification on 14 and 15 pooled samples labeled with dual barcodes at both ends. (mean ± s.e.m.). **(G)** Proportion of singletons of inter-sample chimeras and sequences with correct barcode pairs in 14 and 15 pooled samples. **P < 0.01 (paired t-test).

The antibody repertoire possesses extraordinary diversity. Without encountering the same antigen, the proportion of shared clones (public clones) among different individuals should remain stable (40). However, while investigating repertoires of 83 healthy donors from 14 published Rep-seq data (**Table S1**), we observed that the proportion of intra-project shared clones (antibody clones shared by donors within the same project) was significantly higher than that of inter-project shared clones (antibody clones shared between donors of different projects) (**Figures 1A**, **S1A**). As we reported previously, the number of inter-project shared clones linearly correlated with the product of clone numbers of sample pairs (41). In comparison, the number of intra-project shared clones correlated less well but was much higher than the inter-project ones (**Figure 1B**). In fact, the intra-project clones constituted two thirds (65.07%) of the total shared clones for this healthy repertoire collection (**Figure 1C**). Moreover, we counted the number of singletons, antibody clones with only one supportive read, and found that the proportion of singletons in intra-project shared clones is significantly higher than that in inter-project shared clones (**Figure 1D**). Similarly, the clone fraction of intra-project shared clones is much lower than that of inter-project shared clones (**Figure S1B**). Finally, the clones shared within a project also exhibited a significantly higher frequency of SHMs than did the inter-project ones (**Figure 1E**).

This high frequency of shared clones/reads with high SHMs was intriguing. After careful examination, we hypothesized that these shared clones might be chimeras generated during library preparation, as has been reported in the literature for other HTS applications. Indeed, a test dataset using multiple pooled samples (14 and 15 samples) from the same donor, where each sample was labeled with dual barcodes at both ends and amplified using only six cycles of PCR during library preparation, displayed an average of 12% inter-sample chimeras (**Figure 1F**, *Materials and Methods*). Additionally, the singleton proportion was significantly greater in the inter-sample chimeras than in sequences with correct barcode pairs (**Figure 1G**). The number of SHMs of inter-sample chimeras was also increased, although it is not significantly, relative to that of sequences with correct barcode pairs (**Figure S1C**). These results strongly suggested that the PCR amplification step in library preparation can cause extensive chimeras with an increased singletons and SHMs.

To investigate the contribution of singleton removal for chimera removal, we analyzed the proportion of sequences with correct barcodes and chimeras in singletons and non-singletons respectively. The result showed that about half of the singletons were sequences with correct barcodes, and about 10% of non-singletons were inter-sample chimeras (**Figure S1D**) indicating that removal of singletons can remove a portion of chimeras, but at the same time, some real reads will be discarded. Moreover, nearly two thirds of inter-sample chimeras were non-singletons (**Figures S1E**), which further emphasizes the importance of removing chimeras with barcode pair.

Because these chimeras exhibited normal CDR3s and could be assigned to germline genes *via* analysis tools, they would have been easily mis-identified as inter-sample shared clones resulting from antibody convergence (42) and convergent recombination (43). Furthermore, the nucleotide changes caused by chimera formation would have been recognized as SHMs necessarily introduced during affinity maturation for better binding to antigens. Both of these errors would severely mislead the downstream biological interpretation of repertoire dynamics and the functional study of selected antibodies. Therefore, identification and removal of the chimeras is critical for accurate assessment of the antibody repertoire.

### A Large Proportion of Intra-Sample Chimeras Are Generated by Rep-Seq

Our results above showed that PCR amplification introduced inter-sample chimeras to pooled samples. In the same manner, it could also cause chimeras within a sample. While the inter-sample chimeras in Rep-seq data can be identified and removed by labeling each sample with dual barcodes, identifying and eliminating intra-sample chimeras remained an unresolved issue. To validate and quantify the intra-sample chimeras in Rep-seq data, we designed experiments that amplified antibody sequences from pooled templates of 3, 5, and 10 samples, where each sample was labeled by a different pair of barcodes during pre-amplification using a single GSP (gene-specific primer) (**Figure S2A**). In this design, the reads with incorrect barcode pairs were easily identified as bona fide chimeras. We found that a considerable proportion of Rep-seq reads were chimeras with mis-paired-barcodes (**Figure S2B**). However, this mock Rep-seq using a single GSP may underrepresent the diversity of the antibody repertoire. To mimic a diverse real-world repertoire, we took another sample and separately pre-amplified 8 cycles using 27 GSP pairs, then pooled the products together and amplified with a pair of universal outer primers (**Figure S2A**, *Materials and Methods*). Again, we observed a large proportion of intra-sample chimeras (**Figure S2C**). These results demonstrated that extensive intra-sample chimeras existed in the Rep-seq data and that the extent of chimeras might be affected by the diversities of the input repertoires. However, there were two important shortcomings in these experiments: first, multiple pre-amplification steps may on their own introduce a certain proportion of chimeras; second, the exact sequences in each reaction were not known. These shortcomings impeded our characterization of the critical features of chimeras, such as the frequency of chimera occurrence in different regions of antibodies and the nucleotide errors caused by chimera formation.

**Figure 2:**
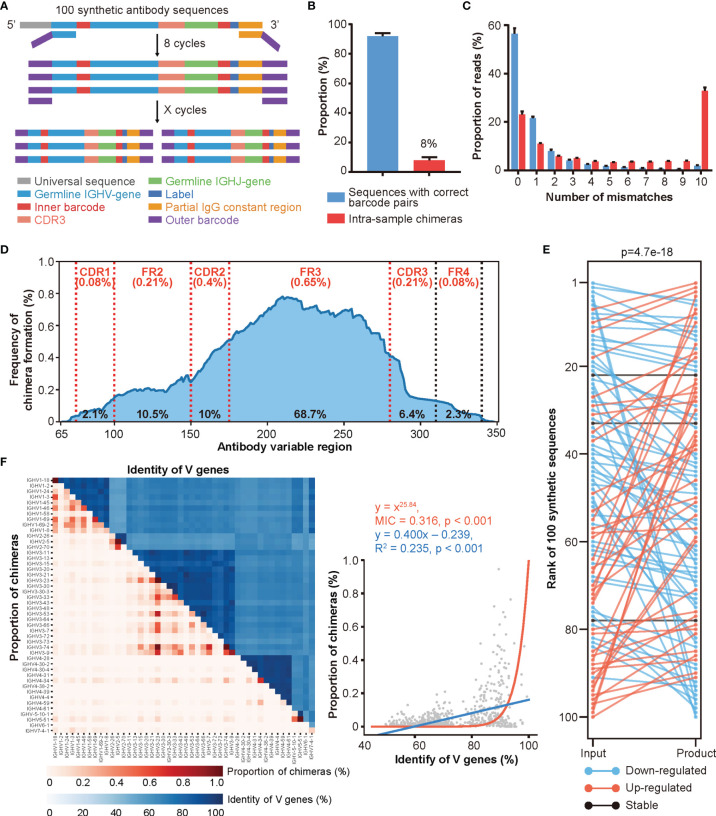
A large proportion of intra-sample chimeras are generated by Rep-seq. **(A)** Schematic diagram of the experimental design for simulating library preparation of an antibody repertoire using 100 synthetic antibody sequences (Experimental Section). **(B)** Distribution of reads with correct and incorrect barcode pairs in Rep-seq reads amplified from 100 synthetic antibody sequences (n = 10). **(C)** Distribution of Rep-seq reads by number of mismatches relative to the best assigned input sequences, for reads with correct and incorrect barcode pairs (n = 10). **(D)** Frequency of chimera formation at each position of V genes. The average and total frequencies of chimera formation in different regions were shown as the top (red) and bottom (black), respectively. **(E)** Changes in clone rank caused by PCR amplification. **(F)** Relationship between the proportions of intra-sample chimeras (red) and the sequence identities (blue) of V gene pairs analyzed using linear and nonlinear models. Forty-six functional V genes are shown in the same order from top to bottom on the Y-axis and from left to right on the X-axis.

To address these issues, we synthesized 100 antibody sequences of various VJ combinations, each with the following design: (i) a universal sequence, (ii) a germline IGHV gene segment with an embedded unique 5’ barcode (B5), (iii) a CDR3 sequence obtained from a real antibody, (iv) a germline IGHJ gene segment, (v) a unique 3’ barcode (B3) followed by a 6 bp label, and (vi) a partial IgG constant region sequence (**Figures 2A**, **S2D**, and **Table S2**). The unique CDR3, B5, and B3 combinations further strengthened our ability to identify and characterize chimeras. To achieve an accurate simulated repertoire, we mixed these synthetic antibodies according to their corresponding V gene usage [as reported previously (41)] and conducted PCR amplification and HTS. We found that approximately 8% of reads represented intra-sample chimeras generated during PCR amplification and sequencing (**Figure 2B**). We then characterized the mismatch information (compared to the original 100 synthetic sequences) for chimeras and the rest of the sequences. The chimera groups showed a higher level of mismatches, which could be misinterpreted as SHMs driven by affinity maturation (**Figure 2C**). Additionally, roughly 20% of chimeras were identical to the synthetic sequences but with incorrect barcode pairs, highlighting the difficulty of identifying chimeras. The sequences with high mismatch numbers in both groups may represent either errors introduced during PCR amplification and/or HTS or multiple iterations of chimeras, as previously reported, due to the low diversity of the original mix (44). Moreover, because we knew the original sequences, we were able to determine the regions of chimera formation (**Figure S2E** and *Materials and Methods*). As shown in **Figure 2D**, the FR3 region showed the highest frequency for chimera formation (with an average of 35.2% frequency of each site within FR3 region located in the breakpoint area and a total of 68.7% of chimeras formed in FR3 region), which was consistent with the previous report (17). After removing the chimeras, we quantified these synthesized sequences and compared them with their input amount. As shown in **Figure 2E**, the quantification of Rep-seq sequences largely disagreed with the input amount indicating that pre-amplification *via* different primer pairs introduces a non-negligible amount of amplification bias.

After validating the intra-sample chimeras and amplification biases, we further characterized the factors that may affect the extent of chimera formation by conducting PCR amplification under different combinations of annealing temperatures, PCR cycles, and amplification methods. Consistent with the low-throughput results reported previously (19, 24, 45, 46), increasing the annealing temperature diminished the formation of chimeras (**Figure S2F**), while increasing the number of amplification cycles promoted the formation of chimeras (**Figure S2G**). Additionally, amplification *via* multiplex PCR led to a slightly higher proportion of chimeras than did amplification using universal primer pairs (**Figure S2H**). Moreover, as shown in **Figure 2F**, V gene groups with higher sequence similarity also correlated with higher proportions of chimeras. Similarly, the FR3 regions of V genes within the same family displayed higher similarities to one another (**Figures S2I, J**), which explained the higher frequency of chimera formation in this region. While we cannot experimentally control antibody sequences, these results suggest that a higher annealing temperature, fewer PCR cycles, and universal primers will reduce the number of chimeras generated by Rep-seq.

### Dumparts Labels Each Molecule With a Unique UMI Pair

**Figure 3:**
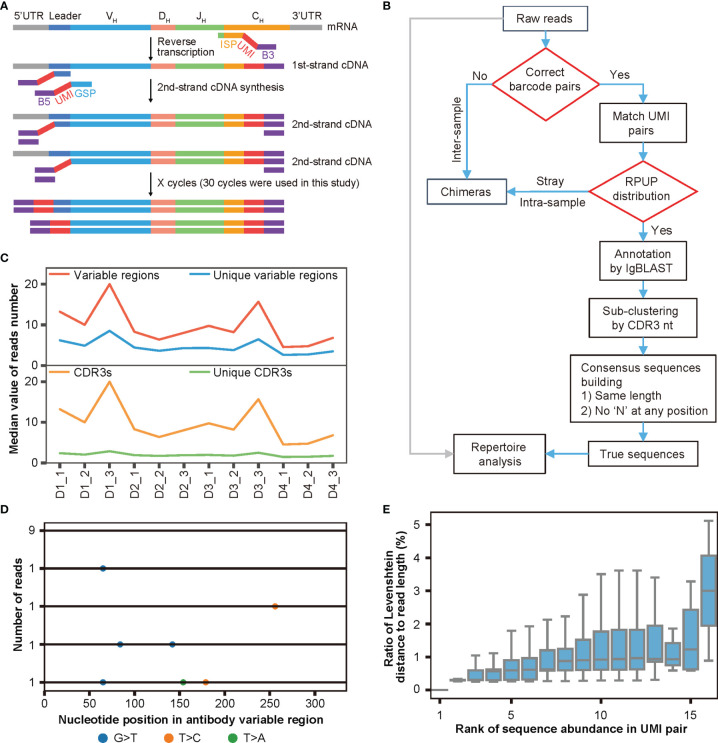
DUMPArts labels each molecule with a unique UMI pair. **(A)** Schematic diagram of DUMPArts for labeling each sample with identical dual barcodes and each molecule with unique dual UMIs. **(B)** Bioinformatics pipeline of DUMPArts for removing chimeras and building consensus sequences. The inter-sample chimeras were removed using dual barcodes during sample splitting, and the intra-sample chimeras were removed using the number of reads per UMI pair (RPUP). **(C)** Number of variable regions, unique variable regions, CDR3s, and unique CDR3s in each UMI pair of Rep-seq data from naïve B cells of 4 donors (D1, D2, D3, and D4). Three replicates from each donor were amplified and analyzed after DUMPArts correction. **(D)** Representative schematic diagram of multiple sequence alignment of the antibody reads in the same UMI pair. The colored dots represent the various types of mismatches. The numbers on the left indicate the abundance of each unique read, and the number on the bottom indicate the base position on the variable region. **(E)** Distribution of ratio of Levenshtein distance to read length with rank of sequence abundance in UMI pair in D2_2. Sequences with lower rank are more abundant in the group. The Levenshtein distance to the most abundant read was calculated for each unique read.

After determining the extent of chimera formation, the adverse effects of chimeras on antibody repertoire analyses, and the factors influencing chimera formation, we decided to develop both experimental strategies and bioinformatics pipelines to remove chimeras from Rep-seq. As shown in **Figure 3A**, we attached a pair of random UMIs and universal primers B5 and B3 (as a barcode pair labeling each sample) to the ends of the cDNA during reverse transcription (RT) and second-strand cDNA synthesis, such that each molecule corresponded to a unique UMI pair. These cDNAs then underwent PCR amplification using a single pair of primers to ensure minimal amplification bias (*Materials and Methods*). For analysis, we identified and removed inter-sample chimeras using the barcode pairs associated with the samples, removed intra-sample chimeras based on the read abundance in each paired UMI group, re-clustered the reads according to both CDR3s and UMI pairs, and built consensus sequences to eliminate nucleotide errors and amplification biases introduced during PCR amplification and HTS (**Figure 3B**, *Materials and Methods*). Thus, our procedure employed dual UMIs with minimal PCR cycles to ensure unique labeling of each molecule for removing artifacts (including intra-sample chimera, nucleotide errors, and amplification biases) and was named DUMPArts.

To avoid the complications of SHM and clonal expansion, we carried out DUMPArts with naïve B cells from 4 donors, each with 3 biological replicates (**Figure S3A**, *Materials and Methods*). To balance the diversity and abundance of UMI pairs, we obtained ultra-deep sequencing for each repertoire. For these 12 samples, we acquired 1.1 million to 20.6 million reads, with a mean of 10 million reads. After grouping by unique UMI pairs and annotating with IgBLAST, the vast majority of the sequences within a UMI pair contain a single unique CDR3 with a limited number of unique variable region sequences (**Figure 3C**). Given that proliferation and mutation are relatively rare in naïve B cells (47, 48), each unique CDR3 should correspond to a single antibody sequence. To ensure the unique labeling of each molecule with a unique pair of UMIs, sequences within a UMI pair were subjected to sub-clustering *via* the CDR3 sequence (**Figure 3B**). Careful examination of the antibody sequences within UMI pairs *via* multiple sequence alignment (MSA) revealed few scattered mismatches (**Figures 3D**, **S3B**), which may represent nucleotide errors introduced during library preparation and HTS. We then calculated the Levenshtein distance of each unique sequence within a UMI pair to the most abundant sequence. We found that less abundant sequences exhibited more mismatches, while the more abundant ones showed fewer mismatches, and the ratio of the Levenshtein distance to the read length of each read within each UMI pair was far below the threshold of 15% reported previously (28) (**Figure 3E**), indicating that sequence reads were derived from the same template RNA after grouping *via* unique UMI pairs and sub-clustering *via* CDR3 nt. Thus, DUMPArts successfully labeled each mRNA molecule with unique pair of UMIs.

### Characterizations of Inter- and Intra-Sample Chimera *via* DUMPArts

**Figure 4:**
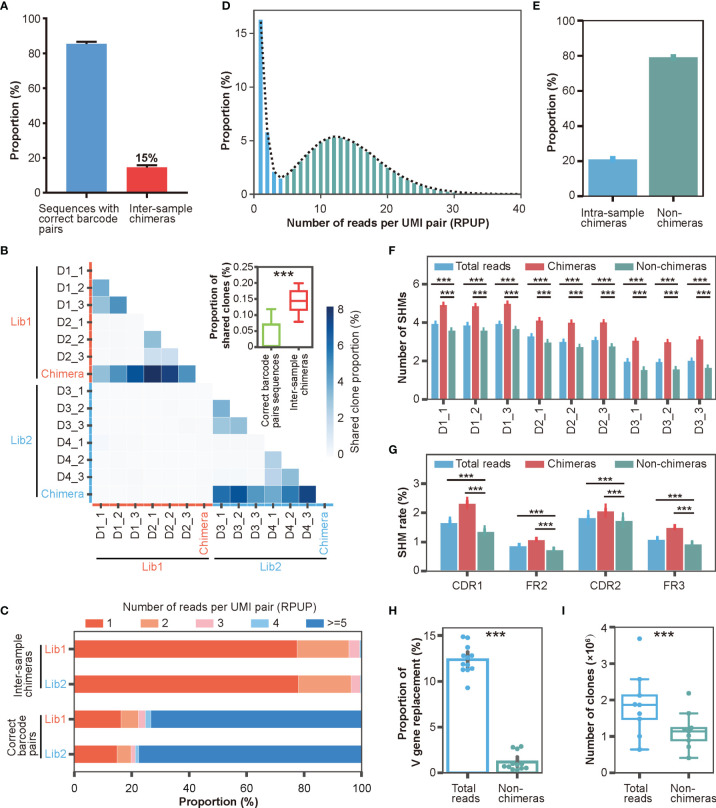
Characterizations of inter- and intra-sample chimera *via* DUMPArts. **(A)** Proportion of inter-sample chimeras in two Rep-seq libraries (Experimental Section). **(B)** Proportion of “shared clones” among samples (n = 12) and inter-sample (n = 12) chimeras. The two separate libraries of four donors (D1, D2, D3, and D4) are shown with red and blue lines on the X- and Y-axes, respectively. The intra-library, inter-sample chimeras were pooled together. ***P < 0.001 (unpaired t-test). **(C)** Distribution of RPUP in both inter-sample chimeras and sequences with correct barcode pairs. **(D)** Proportion distribution of RPUP in sequences with correct barcode pairs. The dotted line represents the trend line of this distribution. **(E)** Proportion of intra-sample chimeras in sequences with correct barcode pairs (n = 12). **(F)** Number of mutations calculated using total reads, intra-sample chimeras, and non-chimeras. **(G)** Mutation rates in the CDR1, FR2, CDR2, and FR3 regions calculated using total reads, intra-sample chimeras, and non-chimeras. ***P < 0.001 (unpaired t-test, mean ± s.e.m.). **(H)** Proportion of V gene replacement in the total reads (n = 12) vs non-chimeras (n = 12). ***P < 0.001 (paired t-test, mean ± s.e.m.). **(I)** Numbers of clones in total reads (n = 9) and non-chimeras (n = 9). ***P < 0.001 (paired t-test).

With the help of DUMPArts’ dual barcode design, we quickly identified and quantified inter-sample chimeras. As shown in **Figure 4A**, the inter-sample chimeras accounted for roughly 15% of the total reads in each library. Consistent with our previous result, these intra-library inter-sample chimeras resulted in a significant proportion of “shared clones” (**Figure 4B**). This, again, indicated that using dual barcodes in each sample is valuable to avoid false discovery of public clones. We also counted the number of singletons and analyzed the proportion of reads that identified as inter-sample chimeras by incorrect barcode pair after computationally removing these singletons. Consistent with the result in **Figure S1D**, even if singletons were removed by bioinformatic processing in advance, there were still about 14% inter-sample chimeras (**Figure S4A**). Moreover, removal of the singletons decreased the SHM number (**Figure S4B**).

Theoretically, chimeras are generated by a random process, and thus, should be relatively rare compared to bona fide antibody sequences in the Rep-seq data. To test this hypothesis, we checked the abundance of inter-sample chimeras and sequences with correct barcode pairs. As shown in **Figure 4C**, inter-sample chimeras were rare, with the vast majority of clones having less than 5 reads per UMI pair (RPUP). In contrast, approximately three-quarters of the sequences with correct barcode pairs had at least 5 RPUPs. This result suggested that low RPUP is a reasonable criterion to remove chimeras. The intra-sample chimeras were generated under the same mechanism as the inter-sample chimeras and therefore should also exhibit very low abundance. We counted the number of UMI pairs per sample (**Figure S4C**) and plotted the RPUP distribution of the reads with correct barcode pairs (**Figure 4D**). Because each RNA molecule was labeled with a unique UMI pair during double-stranded cDNA synthesis, unbiased PCR amplification, and subsequent ultra-deep sequencing, the RPUP values of real sequences and chimeras should conform to a normal distribution and a Poisson distribution, respectively. We therefore removed intra-sample chimeras with Poisson-distributed UMI pairs, accounting for roughly 20% of the sequences with correct barcode (**Figures 4D, E**, and **Table S3**), similar to removing left-skewed k-mers to eliminate sequencing errors for accurate genome assembly (49, 50).

Next, we investigated whether the sequencing depth would affect the ability of DUMPArts to identify the intra-sample chimeras. By randomly sampling the reads at a series of depths (from 1 million to 8 million at intervals of 0.5 million), we found that when sequencing depth is low, especially 1 million, most of the reads are singletons, which conform to a left-skewed distribution and were identified as chimeras. With the increase of sequencing depth, the proportions of intra-sample chimeras identified by DUMPArts were decreased, and the numbers of consensus sequences were increased (**Figure S4D**). These results further emphasized the importance of a greater sequencing depth for obtaining an accurate antibody repertoire.

To illuminate the significance of chimera removal using DUMPArts, we calculated the basic characteristics of the antibody repertoire using total reads and reads without chimeras (non-chimeras). After removal of intra-sample chimeras, the number of mutations in the antibody repertoire was significantly reduced in both the V gene segment (**Figure 4F**) and each separated region (**Figure 4G**), consistent with the low mutation rate in naïve B cells. Furthermore, both V gene and J gene replacement rates were decreased, rectifying the mis-assignment of V gene and J gene caused by chimeras (**Figures 4H**, **S4E–G**). The number of clones was also decreased (**Figures 4I**, **S4H**).

Taken together, these observations suggested that DUMPArts successfully removed inter- and intra-sample chimeras and provided evidence that chimeras generated by Rep-seq can impede the elucidation of a broad spectrum of important antibody repertoire features. In this way, these results highlight the importance of DUMPArts for interpreting molecular information regarding humoral immunity.

### DUMPArts Allows the Acquisition of Accurate Antibody Repertoires

**Figure 5:**
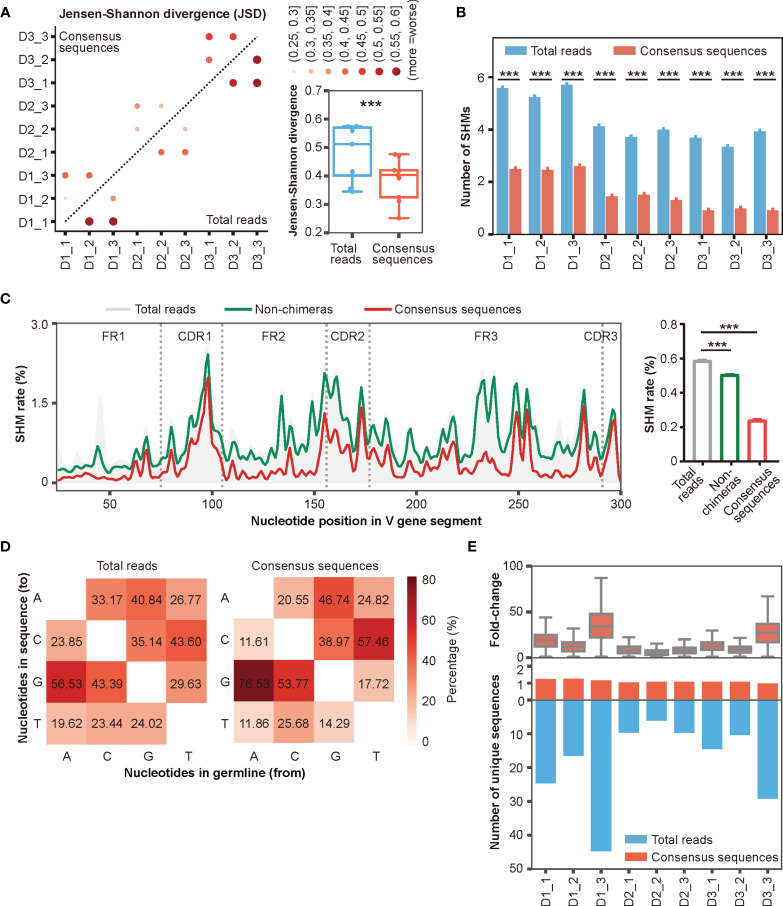
Accurate antibody repertoires are acquired with DUMPArts. **(A)** Jensen-Shannon divergence (JSD) of the top 10 clone compositions in multiple biological replicates calculated using total reads (n = 9) and consensus sequences (n = 9) after DUMPArts correction. ***P < 0.001 (paired t-test). **(B)** Numbers of mutations calculated using total reads and consensus sequences. ***P < 0.001 (unpaired t-test, mean ± s.e.m.). **(C)** Mutation profiling (left) and average mutation rate (right) of IGHV4-39*01 of D3_1 using total reads, non-chimeras, and consensus sequences. X-axis represents the position of the V gene segment, Y-axis represents the mutation rate of each base, and the curves were smoothed by Gaussian filter (left). ***P < 0.001 (unpaired t-test, mean ± s.e.m.). **(D)** Heatmap diagram of the percentages of different mutation patterns from germline V genes (X-axis) to Rep-seq sequences (Y-axis) in D1_1. **(E)** Distribution of the number of unique sequences (lower panel) and their fold-change (upper panel) before and after DUMPArts correction.

In addition to inter-and intra-sample chimeras, another important factor that impedes Rep-seq applications is base errors introduced by PCR and HTS (28, 34, 36–38). These base errors can be eliminated by taking consensus sequences (34, 36). We therefore built consensus sequences for the reads in each UMI pair by taking the most abundant nucleotide at each position (**Figure 3B**, *Materials and Methods*). We then calculated the number of consensus sequences based on the number of unique UMI pairs (**Table S3**). To evaluate the stability of this error correction, we compared the reproducibility of Rep-seq data within multiple biological replicates (grouped by donor) before and after DUMPArts correction. Because the composition of the top clones is one of the most important features of the antibody repertoire, we used the top 10 clone composition as a parameter to calculate the Jensen-Shannon divergence (JSD) between multiple biological replicates. The results showed that DUMPArts error correction improved the similarity among replicates (**Figure 5A**). Similar results were observed using the Morisita–Horn (MH) similarity index calculation (**Figure S5A**).

The improvement provided by error correction was more evident when scrutinizing mutation frequencies in the reads. The number of mutations in consensus sequences was significantly lower than that in the raw reads (**Figure 5B**). Moreover, we observed a stepwise reduction in the number of mutations in IGHV4-39*01, one of the most representative V genes, after chimera removal and consensus sequence building (**Figure 5C**). Notably, DUMPArts correction successfully pruned the erroneous mutations in FR regions. In addition, the nucleotide mutational preference, germline gene usage, top 100 clone distributions, and clonal diversities were also corrected after removing chimeras and building consensus sequences (**Figures 5D**, **S5B–D**). Furthermore, because consensus sequences were built from each UMI pair, the copy number of each unique sequence was decreased by several dozen-fold (**Figure 5E**), indicating that PCR amplification bias has been sufficiently removed from the quantitative analysis of template molecules.

Collectively, these results demonstrated that DUMPArts correction improves the reproducibility of top 10 clone compositions, restores the minimal mutation rate of the naïve B cell repertoire, and eliminates the influence of PCR amplification bias, indicating successful acquisition of an accurate antibody repertoire, which is critical for mAb discovery and antibody-guided vaccine design.

## Discussion

Rep-seq technology has been widely used in the past twenty years and has been successful in several domains, such as the identification of neutralizing mAbs, the design of vaccines, and the tracing of immune history (5–11, 51). However, artifacts intrinsic to the Rep-seq method, such as chimeras, base errors, and amplification biases introduced by the necessary PCR amplification and HTS, have been a longstanding concern. In this study, we describe the development of DUMPArts, which can successfully identify and remove the inter- and intra-sample chimeras, correct base errors and amplification biases, and enable the acquisition of an accurate antibody repertoire.

Chimera is generated when polymerase template-switching on closely-related templates and/or when a partially extended primer anneals with a homologous template to prime the next cycle of DNA synthesis (18). Although it is not the only reason, the number of PCR cycles has been proved to be a critical cause of chimera formation. A thimbleful of chimeras can be arisen at an early PCR cycle, while it will be generated exPLoSively in the later PCR cycles (17–19, 21). Therefore, the copy number of each chimera in the PCR products should be lower than that of a bona fide antibody sequence. Indeed, in our ultra-deep Rep-seq data, almost all the inter-sample chimeras had an RPUP less than 5, and approximately three-quarters of the sequences with correct barcode pairs had an RPUP greater than or equal to 5. Therefore, an appropriate RPUP threshold is critical for removing chimeras and retaining real sequences as much as possible. Previous studies used the strategy of removing singletons or UMI groups with read numbers less than 3 for error correction. Although these methods can remove a portion of chimeras, this ability will be deviated due to the different sequencing depths since most of the reads are singletons when the sequencing depth is 1 million. In this study, we identified the sequences with left-skewed RPUP as intra-sample chimeras, which is of great significance for removing chimeras as much as possible and obtaining real sequences flexibly.

Previous studies using sequential clone hybridization and Sanger sequencing revealed that over 30% of PCR products were chimeras introduced by template switching and PCR-mediated recombination (17–24). But these methods are not applicable for identifying and removing chimeras from Rep-seq data due to the high similarity of V, D, and J genes, and the extraordinary diversity of antibodies. In this study, by constructing dual barcodes and dual UMIs library, we removed the inter-sample chimeras that constitute approximately 15% of library reads, as well as intra-sample chimeras that constitute approximately 20% of the sample reads. Therefore, DUMPArts can identify and remove chimeras from high-throughput Rep-seq data with a precision similar to that of sequential clone hybridization and Sanger sequencing, which indicates the ability of DUMPArts in identifying chimeras.

Preferential recombination (41) and antibody convergence after infection with the same virus (42) bring a subpopulation of shared antibody clonotypes. Upon antigen activation, antibodies undergo SHMs, generating enormous diversity and resulting in better antigen binding affinity (52). Therefore, mutations are essential for the function of antibodies, and the elucidation of the development of antibody clones in turn serves as a guide for vaccine design. However, the relative proportions of shared clones and SHMs are exaggerated due to the existence of chimeras. DUMPArts can help researchers avoid the misidentification of public clones and allows precise identification of SHMs in antibodies for accurate analysis of antibody maturation pathways. This achievement is critical for the elucidation of repertoire dynamics and the identification of functional mAbs. We point out that, in order to ensure the abundance of the UMI pairs, this study used much greater sequencing depth than would common methods. This greater depth is necessary and beneficial for chimera removal, clone and antibody quantification, and error correction.

Using minimal PCR cycles in the RT and second-strand cDNA synthesis step ensures that each molecule is annealed with a unique pair of UMIs. However, this may also cause the loss of rare clones. In addition, the higher abundance threshold for identifying intra-sample chimeras is beneficial for obtaining more credible consensus sequences (29) but eliminates some very low-frequency antibody sequences. Therefore, obtaining more comprehensive antibody repertoires by ameliorating the experimental methods and analytical procedures should be considered for future studies. Taken together, the DUMPArts process efficiently eliminates chimeras and corrects base errors and amplification biases, aids in the acquisition of accurate repertoires with precise antibody sequences, and can facilitate the study of humoral immunity *via* Rep-seq technology.

## Materials and Methods

### Cell Isolation and RNA Extraction

Human peripheral blood was obtained from healthy adult donors and provided by the General Hospital of Southern Theatre Command. All participants provided written informed consent to participate in the study. Peripheral blood mononuclear cells (PBMCs) were isolated from peripheral blood by Ficoll (TBD Science) density-gradient centrifugation. Naïve B cells were isolated from PBMCs using an EasySep Human Naïve B Cell Isolation Kit (17254, STEMCELL Technologies) according to the manufacturer’s protocol. These experiments were performed according to the guidelines of the Research Ethics Committee of Guangdong Provincial People’s Hospital (No.GDREC2020078H(R2)).

### Library Preparation and Sequencing

PBMCs and naïve B cells were subjected to total RNA extraction with the RNeasy Mini Kit (74106, Qiagen) according to the manufacturer’s protocol. For library preparation using DUMPArts, 12 total RNAs from 4 donors were used as templates to synthesize first-strand cDNAs using 10 pmol of each primer in 20-μL reactions (SuperScript II, Thermo Fisher Scientific) using the manufacturer’s protocol and the following thermal cycling program: 42°C for 50 min, 70°C for 15 min. The reaction products were incubated with RNase H at 37°C for 20 min and then purified using 1.2 volumes of AMPure XP beads (Beckman Coulter) and eluted in 25 μL of water. Second-strand cDNAs were synthesized using 23 μL of ssDNA and 10 pmol of each primer in a 50-μL total reaction volume (KAPA HiFi HotStart ReadyMix, Roche). The thermal cycling conditions were as follows: 95°C for 3 min, 98°C for 20 s, 58°C for 20 s, and 72°C for 10 min, with only one cycle. dsDNAs were purified from the PCR product using 0.85 volumes of SPRIselect beads (Beckman Coulter) and eluted in 25 μL of water. VHs were amplified using 22 μL of dsDNA and 10 pmol of each primer in a 50-μL total reaction volume (KAPA HiFi HotStart ReadyMix, Roche) using the following thermal cycling program: 95°C for 3 min; 30 cycles of 98°C for 20 s, 58°C for 15 s, and 72°C for 15 s; and 72°C for 5 min.

For library preparation from pooled templates of 3, 5, and 10 samples, reverse transcription and amplification of total RNA from each sample was carried out using a different pair of barcodes-tagged a single GSP primer pair. For library preparation from pooled templates of 27 PCR products from one sample, reverse transcription and amplification of total RNA was carried out using different pair of barcodes-tagged 27 forward GSP primers and a single reserve GSP primer. The PCR amplification was conducted using the following thermal cycling program: 95°C for 3 min; 8 cycles of 98°C for 20 s, 60°C for 15 s, and 72°C for 15 s; and 72°C for 5 min. Labeled dsDNAs from different samples were pooled as a template for second-round PCR amplification using the following thermal cycling program: 95°C for 3 min; 24 cycles of 98°C for 20 s, 60°C for 15 s, and 72°C for 15 s; and 72°C for 5 min.

PCR products were purified using the NucleoSpin Gel and PCR Clean-up kit (Macherey-Nagel, 704609.250) and subjected to library construction without PCR amplification. For library construction with PCR amplification, the labeled dsDNAs from different samples were pooled and amplified using the following thermal cycling program: 95°C for 3 min; 6 cycles of 98°C for 20 s, 60°C for 15 s, and 72°C for 15 s; and 72°C for 5 min. Libraries were quantified by capillary electrophoresis (Bio-Fragment analyzer, BiOptic Inc.) and pooled accordingly for sequencing using the Illumina platform (MiSeq PE300 and NovaSeq 6000 PE250). All primers are listed in **Table S4**.

### Chimera Elimination

Paired-end FASTQ files acquired from Illumina NovaSeq were merged by PEAR (53) with an minimum overlap length of 20 bp. An in-house Python script was then used to find barcodes and primers. A Levenshtein distance of 1 was used as the threshold to match the barcodes and primers, and any sequences exceeding this threshold were discarded. Reads with barcode pairs that differed from the reference were defined as inter-sample chimeras and removed. The sequence between the barcode and primer was extracted as UMI. These remaining sequences were then grouped by UMI pair, and the number of reads per UMI pair (RPUP) was calculated. The first low ebb of the RPUP distribution was selected as cut-off threshold for identifying intra-sample chimeras. Sequences represented by UMI with RPUP less than the threshold were extracted as intra-sample chimeras and discarded, and sequences represented by the remaining UMIs were subjected to consensus sequence building.

### Consensus Sequence Building

Reads containing the same UMI pair were grouped for consensus sequence building. To correct for rare events in which different molecules were tagged with the same UMI pair, sub-clustering by CDR3 nt was preformed within each UMI pair, and only subgroups contributing to at least 30% of reads in each UMI pair were retained (subgroups with reads equal to 1 were discarded). All reads in a subgroup were then filtered by sequence length, and only those with lengths equal to that of the most abundant sequence were retained. Afterward, a consensus sequence was built for each qualified subgroup, and the final base of each position in the consensus sequence was determined as the most frequent nucleotide. Note that the consensus sequences containing ambiguous most frequent nucleotides (i. e., with equal abundance for the two or more most frequent nucleotides at a position) were discarded.

### Synthetic Sequence Alignment and Breakpoint Inferring

All 100 synthetic antibody sequences were synthesized by GENEWIZ. In brief, each antibody sequence was synthesized, constructed into plasmid, and verified by Sanger sequencing. Then these plasmids were used as templates for PCR amplification, and the PCR products were purified as the final 100 synthetic antibody sequences. All 100 synthetic antibody sequences served as references to build a database for BLAST. Sequencing reads were aligned against the database, and the top aligned read was used to calculate the mismatches, gaps, and alignment lengths. Reads with incorrect B5 and B3 combinations relative to the references were identified as chimeras. To find breakpoints, these chimeras were aligned to the two initial references according to the barcodes. The first mismatches between the initial references and chimera were deemed the bounds, and the overlapping region of the two bounds was identified as the inferred breakpoint area. For the calculation of frequency of chimera formation at each position of V genes, an array with a length equal to the longest sequence was constructed, and the number of times of each position of V genes located in the inferred breakpoint area was calculated. The frequency of chimera formation at each position of V genes was calculated as the number at each position divided by the sum of the array.

### VDJ Annotation

Germline reference sequences were downloaded from IMGT (54), and sequencing reads were aligned to the reference sequences using IgBLAST (55). V(D)J hits, CDR3 nt, alignment length, mismatches, gaps, identity, and BTOP (Blast trace-back operations) information were extracted and stored in a TSV format files *via* an in-house developed Python script. The first 25 bp from the beginning of FR1 and the last 8 bp from the end of FR4 were removed to eliminate possible mismatches induced by primers.

### Antibody Clonotype Analysis

Only sequences with a minimum of 200 bp aligned to V genes were included for clonotype clustering. A clone was defined as having an identical CDR3 sequence and identical V and J gene assignments. Sequencing reads within a clone were grouped, and the clone size was calculated as the number of reads in the clone.

### Somatic Hypermutation Calculation

Details for somatic hypermutation were calculated from the BTOP. The number of somatic hypermutations was calculated for each read, and the SHM of a clone was defined as the average SHM of total reads in that clone. Primer-target regions were not included to avoid possible mismatches induced by primers. The mutation rate of each V segment was calculated using the number of mutations in the reads divided by the alignment length of the V segment. To analyze the mutation at each position of the V gene segment, position information was traced back to the germline, and the mutation frequency at each position for each allele was calculated using the number of sequences with mutations at this position divided by the total number of sequences. The mutation pattern was defined as the ratio of the number of each nucleotide mismatched with germline to the total number of mutated nucleotides.

### Reproducibility Estimation

The Jensen-Shannon divergence (JSD) distance and Morisita-Horn (MH) similarity index were used to estimate the similarity between biological replicates. The top 10 clone compositions were used as parameters to calculate JSD distance and MH similarity index. JSD distance was calculated using the scipy.spatial module (56), and the MH similarity index was calculated using the R package divo (https://CRAN.R-project.org/package=divo).

### V and J Gene Replacement Calculation

All unique sequences with full-length V genes, J genes, and CDR3s were used to analyze V gene replacement. The sequences were grouped by the CDR3 nt sequence, and the number of unique V genes in each group was calculated. CDR3 groups with 2 or more V genes were labeled as V replacement, and the V replacement frequency was calculated as the number of V replacement groups divided by the total number of unique CDR3s. J gene replacement was calculated in the same way.

### The Fold-Change of Unique Sequences Calculation

Sequencing reads were grouped by unique variable regions. The number of unique sequences before correction was calculated from the number of groups with unique variable regions. The number of unique sequences after DUMPArts correction was defined as the number of UMI pairs with unique variable regions. The fold change was calculated as the size before correction divided by the size after correction.

### Statistical Analysis

Statistical analysis was performed *via* two-tailed unpaired and/or paired Student’s t-tests. P-values of <0.05 were considered statistically significant. Box-plot elements are defined (median; whiskers = 1.5; without outliers). Statistics were visualized using GraphPad Prism7.0 or Python3.7.4 *via* the NumPy (57), Pandas (https://pandas.pydata.org), SciPy (56), Seaborn (https://seaborn.pydata.org), and Matplotlib (58) modules.

## Data Availability Statement

The datasets presented in this study can be found in online repositories. The names of the repository/repositories and accession number(s) can be found below: SRA, PRJNA765901.

## Ethics Statement

The studies involving human participants were reviewed and approved by the Research Ethics Committee of Guangdong Provincial People’s Hospital. The patients/participants provided their written informed consent to participate in this study.

## Author Contributions

QW, MW, and HT conducted the biological experiments. HZ, YZ, YFZ, XJY, HL, YC, CM, and CL performed the bioinformatics analyses. CL coordinated the project. ZZ, XQY, and WY conceived the project and designed the biological and computational experiments. QW, HZ, YZ, MW, BL, WY, XQY, and ZZ co-wrote the manuscript. All authors contributed to the article and approved the submitted version.

## Funding

This work was supported by the National Natural Science Foundation of China (NSFC) (31771479, 81991511 and 81991510 to ZZ), NSFC Projects of International Cooperation and Exchanges of NSFC (61661146004 ZZ), and Guangdong-Hong Kong Joint Laboratory Program on Immunological and Genetic Kidney Diseases (2019B121205005 to XQY).

## Conflict of Interest

The authors declare that the research was conducted in the absence of any commercial or financial relationships that could be construed as a potential conflict of interest.

## Publisher’s Note

All claims expressed in this article are solely those of the authors and do not necessarily represent those of their affiliated organizations, or those of the publisher, the editors and the reviewers. Any product that may be evaluated in this article, or claim that may be made by its manufacturer, is not guaranteed or endorsed by the publisher.
