# Supplementary material for: Dual UMIs and Dual Barcodes With Minimal PCR Amplification Removes Artifacts and Acquires Accurate Antibody Repertoire
Source: Front Immunol. 2021 Dec 22;12:778298. doi: 10.3389/fimmu.2021.778298 (PMC8727365; doi:10.3389/fimmu.2021.778298)
Supplement: Supplementary file 1 [file DataSheet_1.docx]

Supplementary Material

# Supplementary Figures and Tables

## Supplementary Figures


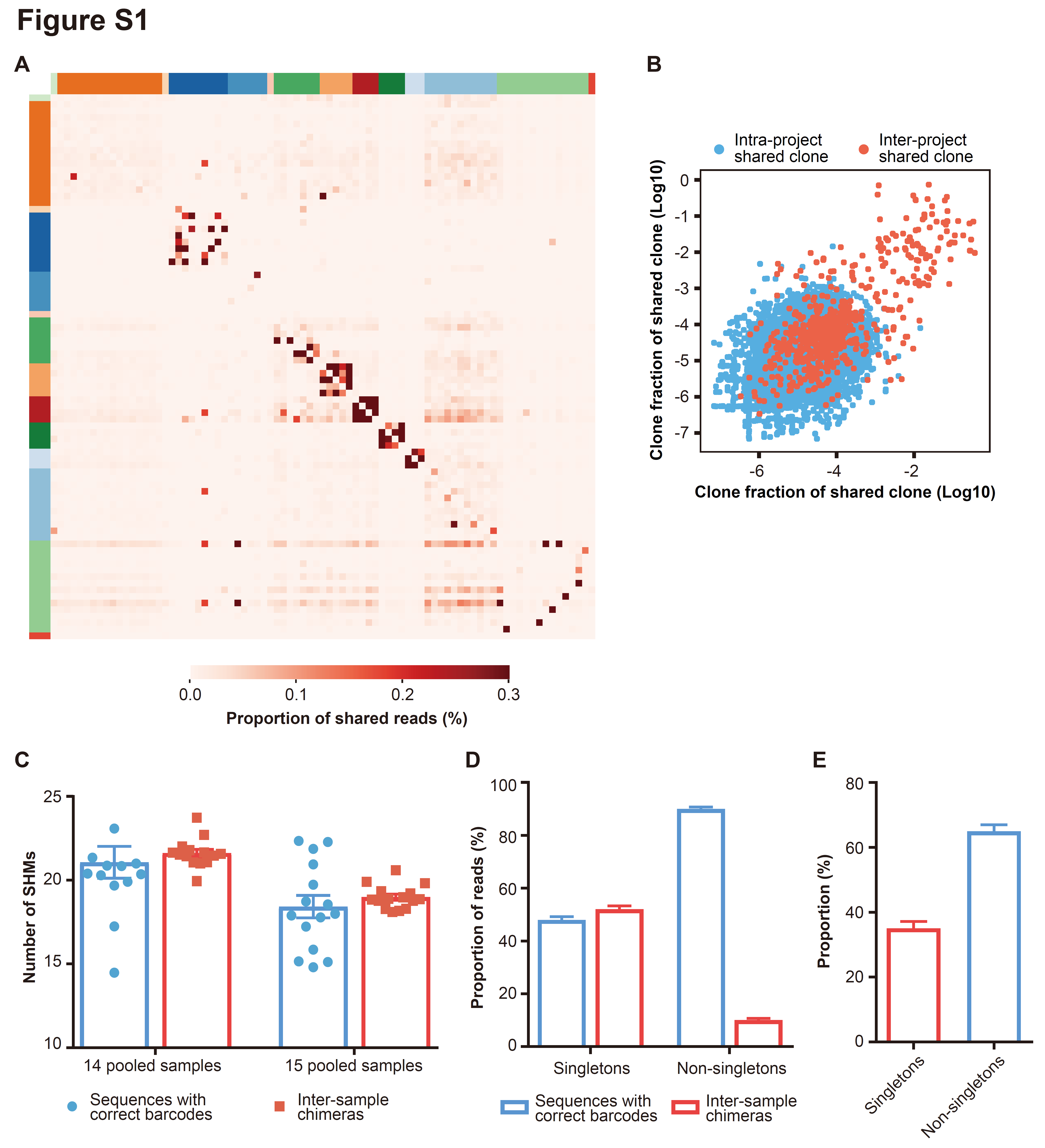


**Supplementary Figure 1. A greater proportion of “shared reads” is present in donors from the same project.** (**A**) Proportion distribution of “shared reads” in 14 published Rep-seq projects. These projects are shown with different colors on the X- and Y-axes; only the Rep-seq data of PBMCs from 83 healthy donors were included. (**B**) Clone fraction of the “shared clones” calculated using sample pair from intra-project and inter-project. (**C**) Number of SHMs of inter-sample chimeras and non-singleton sequences with correct barcode pairs in 14 and 15 pooled samples. (mean ± s.e.m.). (**D**) The proportion of sequences with correct barcodes and chimeras in singletons and non-singletons. (mean ± s.e.m.). (**E**) The distribution of chimeras in singletons and non-singletons. (mean ± s.e.m.).





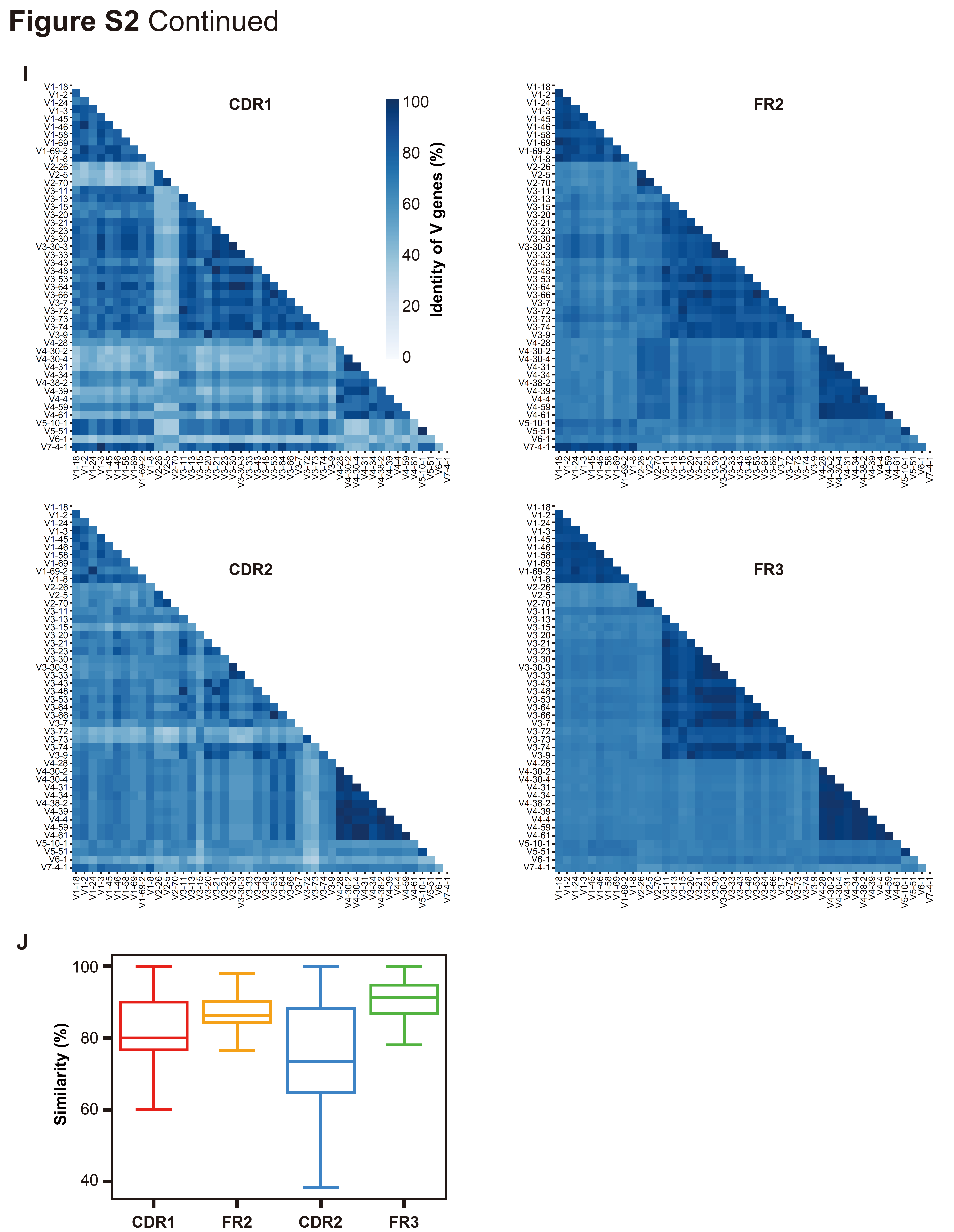


**Supplementary Figure 2. Rep-seq data of pooled antibody repertoires identifies many intra-sample chimeras.** (**A**) Schematic representation of the experimental design for pooling the antibody repertoire from multiple amplification products. The sequences from multiple samples (3, 5, and 10 samples) and one same sample were labeled with different barcode pairs (B5s and B3s) during pre-amplification using a single gene specific primer (GSP) and 27 different GSPs respectively, and the labeled products were pooled for further amplification using outer universal primers. (**B**) Proportions of intra-sample chimeras, defined by the mis-pair of B5 and B3, in the pooled antibody repertoires of 3, 5, and 10 samples (labeled here as “3 mix”, “5 mix”, and “10 mix” respectively) (MATERIALS AND METHODS). (**C**) Proportion of intra-sample chimeras in the pooled antibody repertoire of 27 mixture of pre-amplified antibody sequences (MATERIALS AND METHODS). (**D**) Editing distance between the CDR3 nt sequences of 100 synthetic antibody sequences. (**E**) Diagram of break points inferred by aligning chimeras to corresponding reference sequences. (**F** to **H**) Proportions of chimeras at different annealing temperatures (F, n = 8), PCR cycles (G, n = 4), and amplification methods (H, n = 8). *P < 0.05 and **P < 0.01 (paired t-test, mean ± s.e.m.). (**I**) Pairwise sequence identity of the CDR1, FR2, CDR2, and FR3 region of 100 synthetic antibody sequences. The V genes are shown in the same order from top to bottom on the Y-axis and from left to right on the X-axis. (**J**) Similarities of the CDR1, FR2, CDR2, and FR3 regions on the V genes within the same family (n = 576).


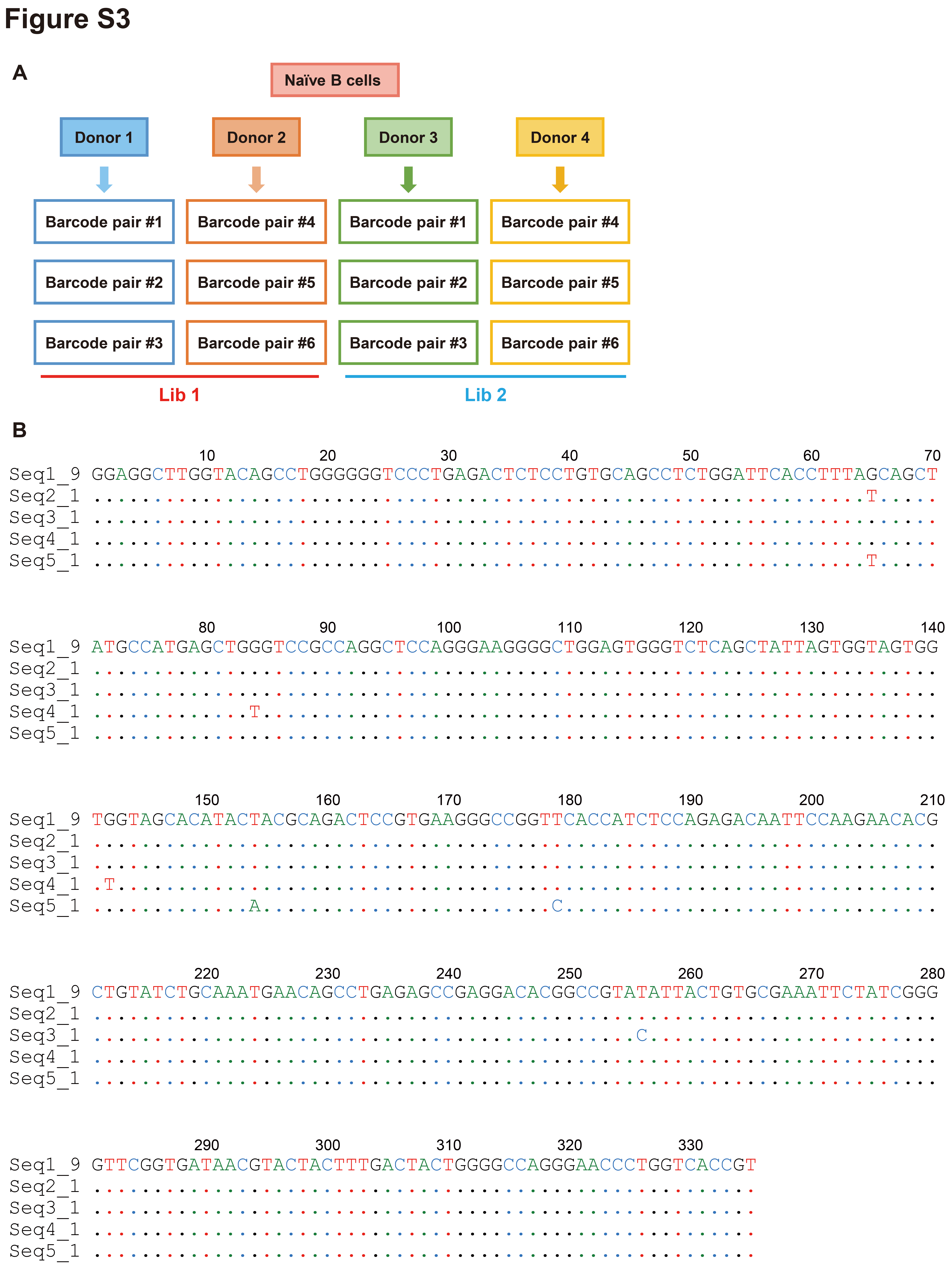


**Supplementary Figure 3. Antibody reads within the same UMI pair are derived from the same template molecule.** (**A**) Schematic showing the library compositions of the 12 samples from 4 donors. Naïve B cells were isolated, and the antibody sequences of each donor were labeled and amplified with 3 different barcode pairs. (**B**) Representative diagram of multiple sequence alignment of the antibody reads in the same UMI pair. The most abundant read was set as the reference. The dots and characters in the other reads represented identical and different bases compared to the reference.


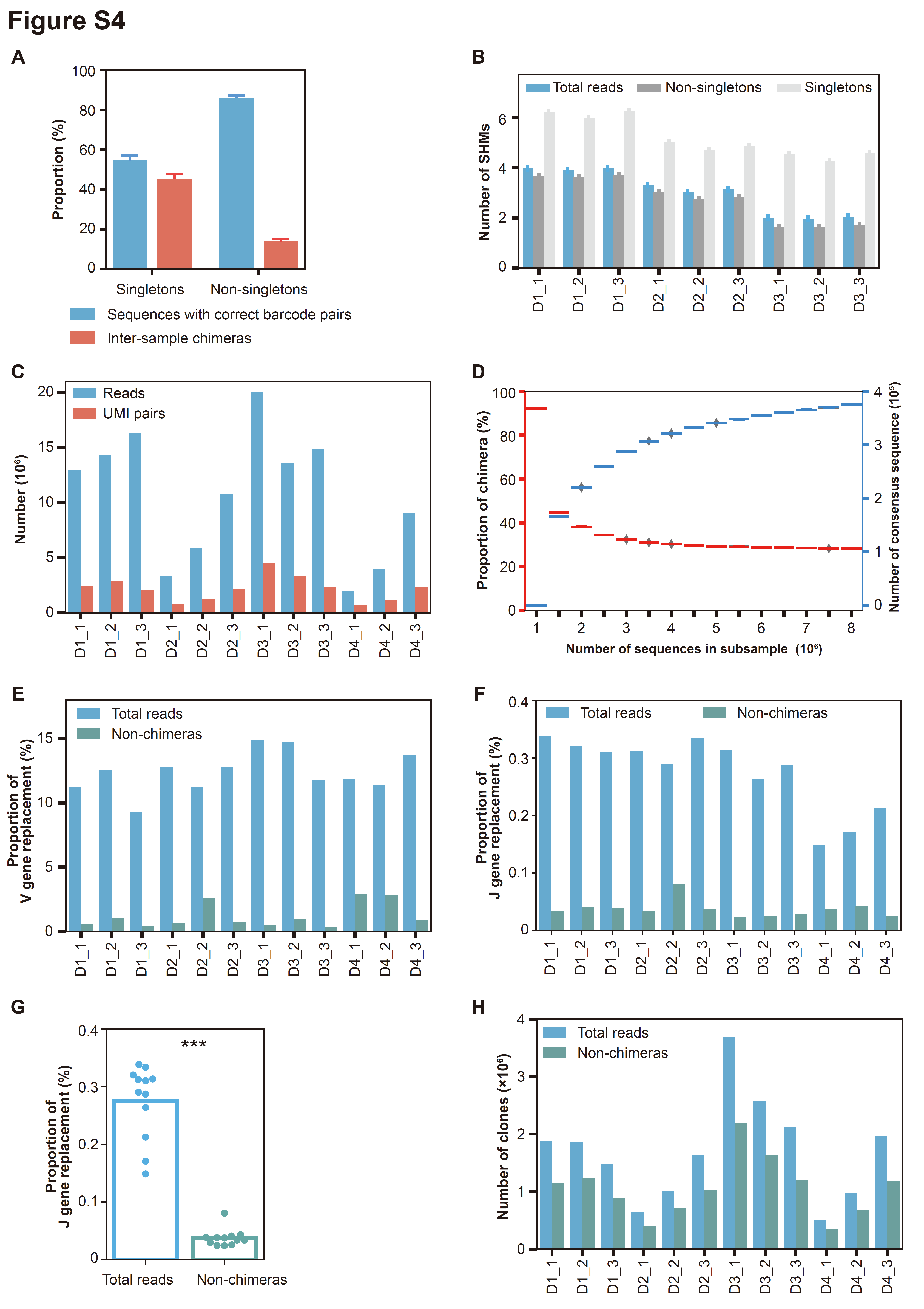


**Supplementary Figure 4.** **Comparison of antibody repertoire characteristics before and after removing chimeras.** (**A**) The proportion of sequences with correct barcodes and chimeras in singletons and non-singletons. (mean ± s.e.m.). (**B**) Number of mutations calculated using total reads, singletons, and non-singletons. ***P < 0.001 (unpaired t-test, mean ± s.e.m.). (**C**) Number of total reads and UMI pairs per sample. (**D**) The proportions of intra-sample chimeras and the numbers of consensus sequences in subsamples. (**E**) Proportion of V gene replacement in total reads and non-chimeras. (**F**) Proportion of J gene replacement in total reads and non-chimeras. (**G**) Statistical analysis of the J gene replacement in total reads (n = 12) and non-chimeras (n = 12). ***P < 0.001 (paired t-test). (**H**) Clone sizes distributions of total reads and non-chimeras.


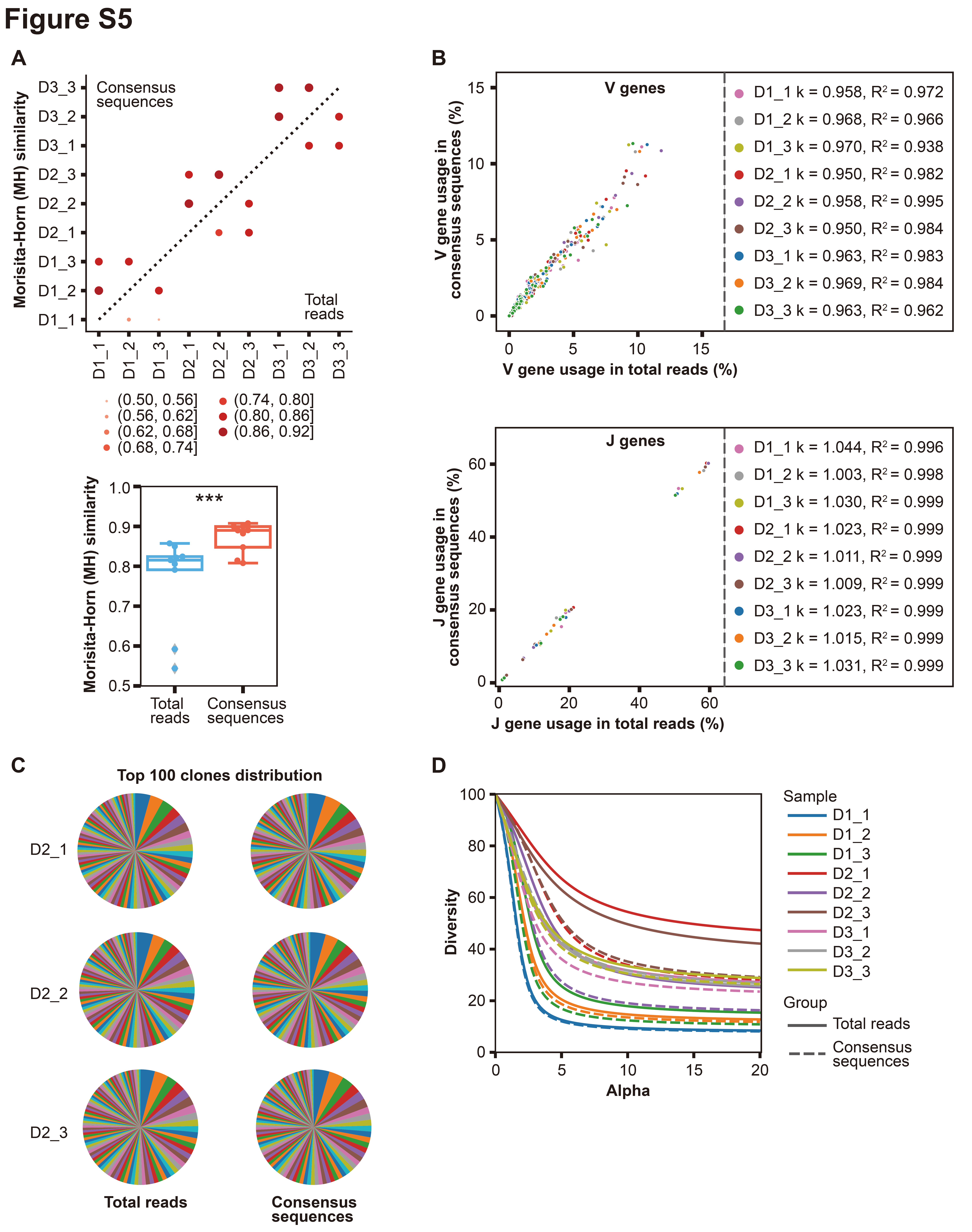


**Supplementary Figure 5. Comparison of antibody repertoire characteristics using total reads and consensus sequences.** (**A**) Morisita–Horn (MH) similarity indices of the top 10 clones in multiple biological replicates calculated using total reads (n = 9) and consensus sequences (n = 9). ***P < 0.001 (paired t-test). (**B**) Correlation of V (upper panel) and J (lower panel) gene usage between total reads (n = 9) and consensus sequences (n = 9). (**C**) Distribution of top 100 clones of total reads and consensus sequences. (**D**) Clonal diversity distribution of total reads (n = 9) and consensus sequences (n = 9).

## Supplementary Tables

| **Supplementary Table 1 Basic information of 83 donors** | | | | | | |
| --- | --- | --- | --- | --- | --- | --- |
| **BioProject** | **Donor** | **Gender** | **Original age** | **Health state** | **Tissue** | **Amplification strategy** |
| PRJEB15295 | Donor 1 | Unknown | Unknown | Healthy | Peripheral blood | RACE PCR |
| PRJEB26509 | Donor 2 | Unknown | Unknown | Healthy | Peripheral blood | RACE PCR |
| PRJEB26509 | Donor 3 | Unknown | Unknown | Healthy | Peripheral blood | RACE PCR |
| PRJEB26509 | Donor 4 | Unknown | Unknown | Healthy | Peripheral blood | RACE PCR |
| PRJEB26509 | Donor 5 | Unknown | Unknown | Healthy | Peripheral blood | RACE PCR |
| PRJEB26509 | Donor 6 | Unknown | Unknown | Healthy | Peripheral blood | RACE PCR |
| PRJEB26509 | Donor 7 | Unknown | Unknown | Healthy | Peripheral blood | RACE PCR |
| PRJEB26509 | Donor 8 | Unknown | Unknown | Healthy | Peripheral blood | RACE PCR |
| PRJEB26509 | Donor 9 | Unknown | Unknown | Healthy | Peripheral blood | RACE PCR |
| PRJEB26509 | Donor 10 | Unknown | Unknown | Healthy | Peripheral blood | RACE PCR |
| PRJEB26509 | Donor 11 | Unknown | Unknown | Healthy | Peripheral blood | RACE PCR |
| PRJEB26509 | Donor 12 | Unknown | Unknown | Healthy | Peripheral blood | RACE PCR |
| PRJEB26509 | Donor 13 | Unknown | Unknown | Healthy | Peripheral blood | RACE PCR |
| PRJEB26509 | Donor 14 | Unknown | Unknown | Healthy | Peripheral blood | RACE PCR |
| PRJEB26509 | Donor 15 | Unknown | Unknown | Healthy | Peripheral blood | RACE PCR |
| PRJEB26509 | Donor 16 | Unknown | Unknown | Healthy | Peripheral blood | RACE PCR |
| PRJEB26509 | Donor 17 | Unknown | Unknown | Healthy | Peripheral blood | RACE PCR |
| PRJNA291102 | Donor 18 | Male | 32 | Healthy | Peripheral blood | Multiplex PCR |
| PRJNA308566 | Donor 19 | Male | 24 | Healthy | Peripheral blood | Multiplex PCR |
| PRJNA308566 | Donor 20 | Female | 25 | Healthy | Peripheral blood | Multiplex PCR |
| PRJNA308566 | Donor 21 | Female | 22 | Healthy | Peripheral blood | Multiplex PCR |
| PRJNA308566 | Donor 22 | Male | 28 | Healthy | Peripheral blood | Multiplex PCR |
| PRJNA308566 | Donor 23 | Female | 33 | Healthy | Peripheral blood | Multiplex PCR |
| PRJNA308566 | Donor 24 | Male | 25 | Healthy | Peripheral blood | Multiplex PCR |
| PRJNA308566 | Donor 25 | Female | 23 | Healthy | Peripheral blood | Multiplex PCR |
| PRJNA308566 | Donor 26 | Female | 38 | Healthy | Peripheral blood | Multiplex PCR |
| PRJNA308566 | Donor 27 | Female | 20 | Healthy | Peripheral blood | Multiplex PCR |
| PRJNA317685 | Donor 28 | Unknown | Unknown | Healthy | Peripheral blood | RACE PCR |
| PRJNA317685 | Donor 29 | Unknown | Unknown | Healthy | Peripheral blood | RACE PCR |
| PRJNA317685 | Donor 30 | Unknown | Unknown | Healthy | Peripheral blood | RACE PCR |
| PRJNA317685 | Donor 31 | Unknown | Unknown | Healthy | Peripheral blood | RACE PCR |
| PRJNA317685 | Donor 32 | Unknown | Unknown | Healthy | Peripheral blood | RACE PCR |
| PRJNA317685 | Donor 33 | Unknown | Unknown | Healthy | Peripheral blood | RACE PCR |
| PRJNA323735 | Donor 34 | Unknown | Unknown | Healthy | Peripheral blood | Multiplex PCR |
| PRJNA324093 | Donor 35 | Unknown | Unknown | Healthy | Peripheral blood | Multiplex PCR |
| PRJNA324093 | Donor 36 | Unknown | Unknown | Healthy | Peripheral blood | Multiplex PCR |
| PRJNA324093 | Donor 37 | Unknown | Unknown | Healthy | Peripheral blood | Multiplex PCR |
| PRJNA324093 | Donor 38 | Unknown | Unknown | Healthy | Peripheral blood | Multiplex PCR |
| PRJNA324093 | Donor 39 | Unknown | Unknown | Healthy | Peripheral blood | Multiplex PCR |
| PRJNA324093 | Donor 40 | Unknown | Unknown | Healthy | Peripheral blood | Multiplex PCR |
| PRJNA324093 | Donor 41 | Unknown | Unknown | Healthy | Peripheral blood | Multiplex PCR |
| Continuted |  |  |  |  |  |  |
| PRJNA335949 | Donor 42 | Unknown | Unknown | Healthy | Peripheral blood | Multiplex PCR |
| PRJNA335949 | Donor 43 | Unknown | Unknown | Healthy | Peripheral blood | Multiplex PCR |
| PRJNA335949 | Donor 44 | Unknown | Unknown | Healthy | Peripheral blood | Multiplex PCR |
| PRJNA335949 | Donor 45 | Unknown | Unknown | Healthy | Peripheral blood | Multiplex PCR |
| PRJNA335949 | Donor 46 | Unknown | Unknown | Healthy | Peripheral blood | Multiplex PCR |
| PRJNA337970 | Donor 47 | Male | 42 | Healthy | Peripheral blood | Multiplex PCR |
| PRJNA337970 | Donor 48 | Unknown | Unknown | Healthy | Peripheral blood | Multiplex PCR |
| PRJNA337970 | Donor 49 | Male | 53 | Healthy | Peripheral blood | Multiplex PCR |
| PRJNA337970 | Donor 50 | Unknown | Unknown | Healthy | Peripheral blood | Multiplex PCR |
| PRJNA338795 | Donor 51 | Male | 33 | Healthy | Peripheral blood | RACE PCR |
| PRJNA338795 | Donor 52 | Male | 33 | Healthy | Peripheral blood | RACE PCR |
| PRJNA338795 | Donor 53 | Male | 31 | Healthy | Peripheral blood | RACE PCR |
| PRJNA338795 | Donor 54 | Female | 31 | Healthy | Peripheral blood | RACE PCR |
| PRJNA349143 | Donor 55 | Male | 30 | Healthy | Peripheral blood | RACE PCR |
| PRJNA349143 | Donor 56 | Male | 55 | Healthy | Peripheral blood | RACE PCR |
| PRJNA349143 | Donor 57 | Male | 32 | Healthy | Peripheral blood | RACE PCR |
| PRJNA381394 | Donor 58 | UNK | 38 | Healthy | Peripheral blood | Multiplex PCR |
| PRJNA381394 | Donor 59 | UNK | 35 | Healthy | Peripheral blood | Multiplex PCR |
| PRJNA381394 | Donor 60 | UNK | 36 | Healthy | Peripheral blood | Multiplex PCR |
| PRJNA381394 | Donor 61 | UNK | 59 | Healthy | Peripheral blood | Multiplex PCR |
| PRJNA381394 | Donor 62 | UNK | 58 | Healthy | Peripheral blood | Multiplex PCR |
| PRJNA381394 | Donor 63 | Unknown | Unknown | Healthy | Peripheral blood | Multiplex PCR |
| PRJNA381394 | Donor 64 | Unknown | Unknown | Healthy | Peripheral blood | Multiplex PCR |
| PRJNA381394 | Donor 65 | Unknown | Unknown | Healthy | Peripheral blood | Multiplex PCR |
| PRJNA381394 | Donor 66 | Unknown | 59 | Healthy | Peripheral blood | Multiplex PCR |
| PRJNA381394 | Donor 67 | Unknown | 57 | Healthy | Peripheral blood | Multiplex PCR |
| PRJNA381394 | Donor 68 | Unknown | 66 | Healthy | Peripheral blood | Multiplex PCR |
| PRJNA393446 | Donor 69 | Unknown | Unknown | Healthy | Peripheral blood | RACE PCR |
| PRJNA393446 | Donor 70 | Unknown | Unknown | Healthy | Peripheral blood | RACE PCR |
| PRJNA393446 | Donor 71 | Unknown | Unknown | Healthy | Peripheral blood | RACE PCR |
| PRJNA393446 | Donor 72 | Unknown | Unknown | Healthy | Peripheral blood | RACE PCR |
| PRJNA393446 | Donor 73 | Unknown | Unknown | Healthy | Peripheral blood | RACE PCR |
| PRJNA393446 | Donor 74 | Unknown | Unknown | Healthy | Peripheral blood | RACE PCR |
| PRJNA393446 | Donor 75 | Unknown | Unknown | Healthy | Peripheral blood | RACE PCR |
| PRJNA393446 | Donor 76 | Unknown | Unknown | Healthy | Peripheral blood | RACE PCR |
| PRJNA393446 | Donor 77 | Unknown | Unknown | Healthy | Peripheral blood | RACE PCR |
| PRJNA393446 | Donor 78 | Unknown | Unknown | Healthy | Peripheral blood | RACE PCR |
| PRJNA393446 | Donor 79 | Unknown | Unknown | Healthy | Peripheral blood | RACE PCR |
| PRJNA393446 | Donor 80 | Unknown | Unknown | Healthy | Peripheral blood | RACE PCR |
| PRJNA393446 | Donor 81 | Unknown | Unknown | Healthy | Peripheral blood | RACE PCR |
| PRJNA393446 | Donor 82 | Unknown | Unknown | Healthy | Peripheral blood | RACE PCR |
| PRJNA395083 | Donor 83 | Female | 23 | Healthy | Peripheral blood | RACE PCR |

| **Supplementary Table 2 CDR3s and starting inputs of 100 synthetic antibody sequences** | | |
| --- | --- | --- |
| **CDR3 ID** | **Sequence** | **Starting inputs (pmol)** |
| 1 | TATGGTTCGTTCTGGGACTGAGGCCTGG | 171.8 |
| 2 | TGTACCGCTATAGGGGCAACTGGGGGG | 4.7 |
| 3 | TGTACAATAAGGGGCTTCGAGTTGAGG | 10.2 |
| 4 | TGTACGGACCCGCCAGCCGGGGTTTGC | 9.2 |
| 5 | TGTGTTACCAGTCCTCCTACGGTTTTT | 3.1 |
| 6 | TGTACCACAGATTTCGGGTCCATCCCC | 11.7 |
| 7 | CCAGGACCGTGCAAGGGCACTGCCTGG | 3.4 |
| 8 | CGTGTGAGGTCGGACAAACACTACTGG | 139.7 |
| 9 | TGCGCGAGAGGGGACTTTGGGGACATCTA | 50.2 |
| 10 | TGTGCGCGGATTGACTGGGCAAGGACTA | 27.4 |
| 11 | TGTCAGCAGTATGGTAGCTCGCACTTTT | 51.8 |
| 12 | TGTTTGGGAGATGGCTACCCCCCGAGA | 50.2 |
| 13 | TGTGCCCTAGGCTATGATAGTAGTTTA | 6.3 |
| 14 | CGTACGCACCTCATGGCGGGATTATTG | 18.5 |
| 15 | TGTGAGGCTGGGCGCACCTACCCTCCTGG | 44.7 |
| 16 | TGTGCGATTTGGCTGGGGTCCTTCGTT | 29.8 |
| 17 | TGTGTATGTATACCACCAGCCCTGACT | 6.1 |
| 18 | ATTTTCGGTTACTTCGATCTCTGG | 4.7 |
| 19 | TGTTTGGGGGATTATCTGGGAATTTGG | 25.9 |
| 20 | TGTGCAACCTCGCAAATAGAGACCTTG | 4.9 |
| 21 | TGTACTAGACAACAAATGCCCCAACCC | 18.8 |
| 22 | CACAGAGAATGATGCGGAGTGG | 41.7 |
| 23 | GCCGTTCAAATAAAAGGAGGTTACTGG | 15.6 |
| 24 | TGTTCCACAGATTTCCCGCGGGGCTTT | 1.8 |
| 25 | CGGCCTTAATGGACTCTGAGTGCTGG | 11.1 |
| 26 | TGCTCCACACAGCCACCCTCTTACTGG | 95.9 |
| 27 | GGCTTTAGCAGCTCGTCCTCGGGG | 27.4 |
| 28 | TGTGTACCCCCCCGAAAACTCAAACTG | 139.7 |
| 29 | TGTGCGAGAGAGAGCCCCCCGACTACTGG | 1.8 |
| 30 | TGTGCGAGTGGGGTCATGTTGACTACGTG | 1.8 |
| 31 | TGTGCTGTCCTTAAGGATCCCCAGTGG | 6.3 |
| 32 | TGCACTCCTTCGGGGTTTGTGGCGTGG | 34.6 |
| 33 | TGCACCACAGGGCGAATAGCAGCTGCT | 2.5 |
| 34 | TGTGTTTGTCTGGATCAAGATGAATGG | 34.6 |
| 35 | TGCGTTCATAGTAATGGTTACTTTTGG | 2.5 |
| 36 | TGTTCTTATGACTACGGTGAGAGGATGG | 51.8 |
| 37 | TGTGAGACTACAACGATATTAACTACTGG | 10.0 |
| 38 | TGTGCGAAATCCTCCCTGAGCACTTGC | 33.3 |
| 39 | TGTTATCGGTCCGGCGGGAGTATGTGG | 15.6 |
| 40 | TGTTTGAGATATGATGTCCTTATGTTTGG | 18.2 |
| 41 | TATGGTTCGGGGA | 6.1 |
| 42 | TGTATGTGCTATACTTCGGGGGCTTCG | 3.4 |
| 43 | TCTGCGAAAAAACTGAGTCATGCGGCACG | 1.8 |
| 44 | GGGATCTGGCAGGTGTCGCTTCCTTCTGG | 13.0 |
| 45 | TGTACGAAAAACGTGGTTACTGCTTCTGG | 44.7 |
| 46 | TAGCAGTGGCTGGTACTCTGCCTACTGG | 18.5 |
| 47 | GCAGTGGCTACGAGAAGTGGGCTGGTG | 29.8 |
| Continued |  |  |
| 48 | TGTATAAGAGGGGATTGGTACCATCTGG | 13.2 |
| 49 | TGTGTGATCAACCAGATCGCGGCAGGG | 10.2 |
| 50 | GCCCGACTATGGGGATCTTTGACTACCGG | 79.9 |
| 51 | TATGCAAGGTCATTTGGACAGCTCGTTTG | 50.6 |
| 52 | TGTATGAATGCAGAGGGGGAATTCAAG | 18.5 |
| 53 | TGTTCGACCTATTCATTGGGCTGG | 2.5 |
| 54 | TGTTTTGGGGGGAGCGTCCCATAT | 57.8 |
| 55 | TGTGCACCTCCAAACCCCGCCCCGCTGG | 3.4 |
| 56 | GATATTTTGACTGGTTACGTAGGA | 4.9 |
| 57 | TGTGCGGATCTGAGTTACAGCGACTGC | 15.6 |
| 58 | GTGCGAGATTCTTGGCCCCAGACGTCGAG | 18.8 |
| 59 | TGTATGAAATTACAGGGAGTTTCGCTC | 3.3 |
| 60 | TGTTTATTAACGACTACATTGACCGCTAG | 6.0 |
| 61 | CGTGCTCATTTCCGTTCGGGGACTTCA | 15.3 |
| 62 | GGGGGGCTGACTGGTACTTCGATCTCTGG | 11.7 |
| 63 | TGTGCCCCCCTACCCAAAATCGGG | 13.2 |
| 64 | TGTGGTCCGGGTATAGCAGCACTCGGG | 16.8 |
| 65 | TGCTCGGAGATGTCTACAATGACCCCGG | 11.7 |
| 66 | TGTGCACACTCGGGCATACTGCTTACT | 50.6 |
| 67 | TTCTTATAGGTAGTGGGTAGGTCGGAG | 29.8 |
| 68 | CGTGCCACATGGTCCGGGAGTGCCGTA | 12.7 |
| 69 | TGTAAGGTGAGCTTTGGGTATTAAGGTGG | 62.3 |
| 70 | TGTTTAAGCCCAAAATTAGGCTACTGG | 51.8 |
| 71 | TGTACTATCGACGTTCATTCCCTTTGG | 50.6 |
| 72 | TGCGCGAAAGGTGATATTGATATTAAG | 6.0 |
| 73 | TGCGCGGTCGGGTGGTTCGACCCCTGG | 6.3 |
| 74 | TGTGCGGAAGCCCACGGCGGATGGCCC | 92.0 |
| 75 | CCGGCAGCAGCCCTAGACTATTGG | 34.6 |
| 76 | TGTGCGGGTTCAAACTGGAACGCC | 3.1 |
| 77 | TGTTTGAGCCATGTCCAGCTTCCATCTGG | 1.8 |
| 78 | TGGGCTGGTGCTTATTAATAGACTACTGG | 44.7 |
| 79 | TGTTCCCGATCGACCGCAGGCCGGTGG | 16.8 |
| 80 | TGTGCGGGTTCGATATTATACTAATGGCG | 92.0 |
| 81 | TGTGTGGCTATAGGAACAGCCGGCCCTGG | 57.8 |
| 82 | TGAGGGAGACATATGTCCGGGCACAGAAG | 10.0 |
| 83 | TGTGCGACAAGATTCCAGTATTGGATC | 10.0 |
| 84 | TGCGTTAAAGACCCCACTGCGAACTGG | 171.8 |
| 85 | TGTGGCGTCATTGCAAACTTTGACTACT | 18.8 |
| 86 | TGTTCCCAAGTCGAGATGGCCACC | 62.3 |
| 87 | TGTGCAAGTCATGGCGGGCCGGGGATG | 3.4 |
| 88 | TGTACGAGGATCCCCGATTACTTCGGG | 120.4 |
| 89 | TGTATCACATCTCCAGTATGGTAGACCGG | 27.4 |
| 90 | TATGCGGAATCTTGTACTAAATACAGG | 13.0 |
| 91 | TGCACGTGAACCACATTTTTCCGACTGG | 6.1 |
| 92 | TGTGCGAGAGAGGTGGACAACAATAGCT | 5.7 |
| 93 | TGTGTGTGTACACTGGCTGGTACGATA | 3.4 |
| 94 | TGTGCTAATTGGGGGGGCTGGGCTCCTGG | 4.9 |
| 95 | TGTGTAACTTATTACTATGGTTTTGGC | 4.7 |
| 96 | AGCTCACGTCTATCCCGACTACTGG | 3.3 |
| 97 | CCCTATGAGACTAGTGATTCTTAAATTGG | 9.2 |
| Continued |  |  |
| 98 | TGTGTCCTGCTGCTCCACTTGACTCCTGG | 1.8 |
| 99 | TGTTTGTCGGGGCCTGCTGGCTCG | 197.4 |
| 100 | TGGCGTCGCCTGGAACTTCGGGACA | 3.3 |

| **Supplementary Table 3 Number of sequences** | | | | |
| --- | --- | --- | --- | --- |
| **Sample** | **Total reads** | **Chimeras** | **Non-chimeras** | **Consensus sequences** |
| D1_1 | 11,463,363 | 2,222,317 | 8,357,151 | 376,819 |
| D1_2 | 10,687,526 | 2,297,381 | 8,365,037 | 573,736 |
| D1_3 | 10,733,166 | 1,670,860 | 8,050,914 | 191,944 |
| D2_1 | 2,843,474 | 591,016 | 2,079,478 | 197,426 |
| D2_2 | 3,912,567 | 601,124 | 3,038,589 | 457,126 |
| D2_3 | 7,204,681 | 1,464,819 | 5,161,155 | 509,075 |
| D3_1 | 18,671,185 | 4,170,035 | 13,658,419 | 914,634 |
| D3_2 | 12,143,408 | 2,601,830 | 9,099,779 | 854,758 |
| D3_3 | 12,892,216 | 2,423,684 | 9,279,082 | 289,389 |
| D4_1 | 1,765,021 | 354,050 | 1,303,588 | 276,289 |
| D4_2 | 3,188,211 | 549,858 | 2,418,997 | 495,707 |
| D4_3 | 8,282,849 | 1,894,369 | 5,731,806 | 670,388 |

| **Supplementary Table 4 Primers used in the study** | | |
| --- | --- | --- |
| **Primers used in simulated antibody repertoire** | | |
| **Primer ID** | | **Sequence** |
| P5S1 | | ACGACTAGCTATGAGCAGAAGCCTGGGTCCTCGG |
| P5S2 | | ACGACTAGCTTAGGCTGAGGTGCAGCTGGTGGAG |
| P5S3 | | ACGACTAGCTGATTCCAAAAGCCCGGGGAGTCTC |
| P5S4 | | ACGACTAGCTGTAACTACCTTCAGTGACCACTAC |
| P5S5 | | ACGACTAGCTCACTCACAGGTCACCTTGAAGGAG |
| P5S6 | | ACGACTAGCTCATGGTTGCGCTGGTGAAACCCAC |
| P5S7 | | ACGACTAGCTCCAACTGCTCCATCAGCAGTAGTA |
| P5S8 | | ACGACTAGCTCTCAGAGAGGTGCAGCTGTTGGAG |
| P5S9 | | ACGACTAGCTAATCGGCAGGTGCAGCTGTTGGAG |
| P5S10 | | ACGACTAGCTTACTGCCAGGTGCAGCTGGTGGAC |
| P5S11 | | ACGACTAGCTCGATCACAAATGCAGCTGGTGCAG |
| P5S12 | | ACGACTAGCTCCTTGATCTGGTGGCTCCGTCAGC |
| P5S13 | | ACGACTAGCTAGCTTACAGGTGCAGCTACAGGAG |
| P5S14 | | ACGACTAGCTTGTAGGCAGGTCCAGCTTGTGCAG |
| P5S15 | | ACGACTAGCTATCACGCAGGTCCAGCTGGTACAG |
| P5S16 | | ACGACTAGCTTTAGGCCTCTGGTGGCTCCATCAG |
| P5S17 | | ACGACTAGCTACAGTGGAGGTCCAGCTGGTACAG |
| P5S18 | | ACGACTAGCTCAGATCTATGGTGGGTCCTTCAGT |
| P5S19 | | ACGACTAGCTGATCAGTCTGGTGGCTCCATCAGT |
| P5S20 | | ACGACTAGCTTCTCACCAGGTGCAGCTGGTGGAG |
| Continued | |  |
| P5S21 | | ACGACTAGCTGTCGATCAGGTGCAGCTGGTGCAA |
| P5S22 | | ACGACTAGCTCTAGTGCGGGTCACCTTGAGGGAG |
| P5S23 | | ACGACTAGCTGAGTTGCAGGTGCAGCTGGTGCAG |
| P5S24 | | ACGACTAGCTGGTACAGCTGGTGAAACCCACACA |
| P5S25 | | ACGACTAGCTAAGGTCAGAAGCCTGGGGCTACAG |
| P5S26 | | ACGACTAGCTACGAGTGAGGTACAACTGGTGGAG |
| P5S27 | | ACGACTAGCTATTCCGCAGGTGCAGCTACAACAG |
| P3S1 | | ACTCGAAGTTCAGTCGTACGCAGGGGAAGACCGATGGGCCCTTGGTGG |
| P3S2 | | ACTCGAAGTTCAGTCGTAAGCGGGGGAAGACCGATGGGCCCTTGGTGG |
| P3S3 | | ACTCGAAGTTCAGTCGTAGGTCGGGGAAGACCGATGGGCCCTTGGTGG |
| P3S4 | | ACTCGAAGTTCAGTCGAGGGTAGGGGAAGACCGATGGGCCCTTGGTGG |
| P3S5 | | ACTCGAAGTTCAGTCGTAGGCTGGGGAAGACCGATGGGCCCTTGGTGG |
| P3S6 | | ACTCGAAGTTCAGTCGCGATCAGGGGAAGACCGATGGGCCCTTGGTGG |
| P3S7 | | ACTCGAAGTTCAGTCGGCTACTGGGGAAGACCGATGGGCCCTTGGTGG |
| P3S8 | | ACTCGAAGTTCAGTCGCAGTGAGGGGAAGACCGATGGGCCCTTGGTGG |
| P3S9 | | ACTCGAAGTTCAGTCGGTAGACGGGGAAGACCGATGGGCCCTTGGTGG |
| P3S10 | | ACTCGAAGTTCAGTCGCGATTGGGGGAAGACCGATGGGCCCTTGGTGG |
| P3S11 | | ACTCGAAGTTCAGTCGACTCGTGGGGAAGACCGATGGGCCCTTGGTGG |
| P3S12 | | ACTCGAAGTTCAGTCGCTCATGGGGGAAGACCGATGGGCCCTTGGTGG |
| P3S13 | | ACTCGAAGTTCAGTCGAAGGAGGGGGAAGACCGATGGGCCCTTGGTGG |
| P3S14 | | ACTCGAAGTTCAGTCGGTACTAGGGGAAGACCGATGGGCCCTTGGTGG |
| P3S15 | | ACTCGAAGTTCAGTCGATGCGAGGGGAAGACCGATGGGCCCTTGGTGG |
| P3S16 | | ACTCGAAGTTCAGTCGTCTCTCGGGGAAGACCGATGGGCCCTTGGTGG |
| P3S17 | | ACTCGAAGTTCAGTCGACTGGAGGGGAAGACCGATGGGCCCTTGGTGG |
| P3S18 | | ACTCGAAGTTCAGTCGGAGTTCGGGGAAGACCGATGGGCCCTTGGTGG |
| P3S19 | | ACTCGAAGTTCAGTCGTAGCCTGGGGAAGACCGATGGGCCCTTGGTGG |
| P3S20 | | ACTCGAAGTTCAGTCGCCTAGAGGGGAAGACCGATGGGCCCTTGGTGG |
| P3S21 | | ACTCGAAGTTCAGTCGCTGTACGGGGAAGACCGATGGGCCCTTGGTGG |
| P3S22 | | ACTCGAAGTTCAGTCGAAGGTTGGGGAAGACCGATGGGCCCTTGGTGG |
| P3S23 | | ACTCGAAGTTCAGTCGATGCAAGGGGAAGACCGATGGGCCCTTGGTGG |
| P3S24 | | ACTCGAAGTTCAGTCGGTCTCTGGGGAAGACCGATGGGCCCTTGGTGG |
| P3S25 | | ACTCGAAGTTCAGTCGTCCTGTGGGGAAGACCGATGGGCCCTTGGTGG |
| P3S26 | | ACTCGAAGTTCAGTCGTATCGCGGGGAAGACCGATGGGCCCTTGGTGG |
| P3S27 | | ACTCGAAGTTCAGTCGTCGACAGGGGAAGACCGATGGGCCCTTGGTGG |
| 2nd P5 Primer | | CATGGACCTTACGACTAGCT |
| 2nd P3 Primer | | GACTAGGAACTCGAAGTTCAGTCG |
| **Primers used in DUMPArts** | | |
| **Primer ID** | **Sequence** | |
| B1-F1 | CGCCAAGTGTCTATGCNNNNNNNNCAGGTCACCTTGAGGGAG | |
| B1-F2 | CGCCAAGTGTCTATGCNNNNNNNNCAGGTGCAGCTGGTGCAG | |
| B1-F3 | CGCCAAGTGTCTATGCNNNNNNNNCAGGTGCAGCTACAGCAGTGGG | |
| B1-F4 | CGCCAAGTGTCTATGCNNNNNNNNCAGGTGCAGCTGCAGGAGTCGG | |
| B1-F5 | CGCCAAGTGTCTATGCNNNNNNNNCAGGTCCAGCTGGTGCAGTCTGG | |
| B1-F6 | CGCCAAGTGTCTATGCNNNNNNNNTTAAAAGGTGTCCAGTGTGAGG | |
| B1-F7 | CGCCAAGTGTCTATGCNNNNNNNNCAGGTACAGCTGCAGCAGTCA | |
| B1-F8 | CGCCAAGTGTCTATGCNNNNNNNNCAGGTGCAGCTGGTGGAGTCTGG | |
| B1-F9 | CGCCAAGTGTCTATGCNNNNNNNNCAACTACAGGTGCCCACTCC | |
| B1-F10 | CGCCAAGTGTCTATGCNNNNNNNNGAGGTGCAGCTGGTGGAGTCT | |
| B2-F1 | TGAGCGGATAACATGCNNNNNNNNCAGGTCACCTTGAGGGAG | |
| Continued |  | |
| B2-F2 | TGAGCGGATAACATGCNNNNNNNNCAGGTGCAGCTGGTGCAG | |
| B2-F3 | TGAGCGGATAACATGCNNNNNNNNCAGGTGCAGCTACAGCAGTGGG | |
| B2-F4 | TGAGCGGATAACATGCNNNNNNNNCAGGTGCAGCTGCAGGAGTCGG | |
| B2-F5 | TGAGCGGATAACATGCNNNNNNNNCAGGTCCAGCTGGTGCAGTCTGG | |
| B2-F6 | TGAGCGGATAACATGCNNNNNNNNTTAAAAGGTGTCCAGTGTGAGG | |
| B2-F7 | TGAGCGGATAACATGCNNNNNNNNCAGGTACAGCTGCAGCAGTCA | |
| B2-F8 | TGAGCGGATAACATGCNNNNNNNNCAGGTGCAGCTGGTGGAGTCTGG | |
| B2-F9 | TGAGCGGATAACATGCNNNNNNNNCAACTACAGGTGCCCACTCC | |
| B2-F10 | TGAGCGGATAACATGCNNNNNNNNGAGGTGCAGCTGGTGGAGTCT | |
| B3-F1 | AAGCTGCGGAATATGCNNNNNNNNCAGGTCACCTTGAGGGAG | |
| B3-F2 | AAGCTGCGGAATATGCNNNNNNNNCAGGTGCAGCTGGTGCAG | |
| B3-F3 | AAGCTGCGGAATATGCNNNNNNNNCAGGTGCAGCTACAGCAGTGGG | |
| B3-F4 | AAGCTGCGGAATATGCNNNNNNNNCAGGTGCAGCTGCAGGAGTCGG | |
| B3-F5 | AAGCTGCGGAATATGCNNNNNNNNCAGGTCCAGCTGGTGCAGTCTGG | |
| B3-F6 | AAGCTGCGGAATATGCNNNNNNNNTTAAAAGGTGTCCAGTGTGAGG | |
| B3-F7 | AAGCTGCGGAATATGCNNNNNNNNCAGGTACAGCTGCAGCAGTCA | |
| B3-F8 | AAGCTGCGGAATATGCNNNNNNNNCAGGTGCAGCTGGTGGAGTCTGG | |
| B3-F9 | AAGCTGCGGAATATGCNNNNNNNNCAACTACAGGTGCCCACTCC | |
| B3-F10 | AAGCTGCGGAATATGCNNNNNNNNGAGGTGCAGCTGGTGGAGTCT | |
| B4-F1 | ACGCCATATCGCATGCNNNNNNNNCAGGTCACCTTGAGGGAG | |
| B4-F2 | ACGCCATATCGCATGCNNNNNNNNCAGGTGCAGCTGGTGCAG | |
| B4-F3 | ACGCCATATCGCATGCNNNNNNNNCAGGTGCAGCTACAGCAGTGGG | |
| B4-F4 | ACGCCATATCGCATGCNNNNNNNNCAGGTGCAGCTGCAGGAGTCGG | |
| B4-F5 | ACGCCATATCGCATGCNNNNNNNNCAGGTCCAGCTGGTGCAGTCTGG | |
| B4-F6 | ACGCCATATCGCATGCNNNNNNNNTTAAAAGGTGTCCAGTGTGAGG | |
| B4-F7 | ACGCCATATCGCATGCNNNNNNNNCAGGTACAGCTGCAGCAGTCA | |
| B4-F8 | ACGCCATATCGCATGCNNNNNNNNCAGGTGCAGCTGGTGGAGTCTGG | |
| B4-F9 | ACGCCATATCGCATGCNNNNNNNNCAACTACAGGTGCCCACTCC | |
| B4-F10 | ACGCCATATCGCATGCNNNNNNNNGAGGTGCAGCTGGTGGAGTCT | |
| B5-F1 | TATAAGCAGAGCATGCNNNNNNNNCAGGTCACCTTGAGGGAG | |
| B5-F2 | TATAAGCAGAGCATGCNNNNNNNNCAGGTGCAGCTGGTGCAG | |
| B5-F3 | TATAAGCAGAGCATGCNNNNNNNNCAGGTGCAGCTACAGCAGTGGG | |
| B5-F4 | TATAAGCAGAGCATGCNNNNNNNNCAGGTGCAGCTGCAGGAGTCGG | |
| B5-F5 | TATAAGCAGAGCATGCNNNNNNNNCAGGTCCAGCTGGTGCAGTCTGG | |
| B5-F6 | TATAAGCAGAGCATGCNNNNNNNNTTAAAAGGTGTCCAGTGTGAGG | |
| B5-F7 | TATAAGCAGAGCATGCNNNNNNNNCAGGTACAGCTGCAGCAGTCA | |
| B5-F8 | TATAAGCAGAGCATGCNNNNNNNNCAGGTGCAGCTGGTGGAGTCTGG | |
| B5-F9 | TATAAGCAGAGCATGCNNNNNNNNCAACTACAGGTGCCCACTCC | |
| B5-F10 | TATAAGCAGAGCATGCNNNNNNNNGAGGTGCAGCTGGTGGAGTCT | |
| B6-F1 | CGCCGACATCATATGCNNNNNNNNCAGGTCACCTTGAGGGAG | |
| B6-F2 | CGCCGACATCATATGCNNNNNNNNCAGGTGCAGCTGGTGCAG | |
| B6-F3 | CGCCGACATCATATGCNNNNNNNNCAGGTGCAGCTACAGCAGTGGG | |
| B6-F4 | CGCCGACATCATATGCNNNNNNNNCAGGTGCAGCTGCAGGAGTCGG | |
| B6-F5 | CGCCGACATCATATGCNNNNNNNNCAGGTCCAGCTGGTGCAGTCTGG | |
| B6-F6 | CGCCGACATCATATGCNNNNNNNNTTAAAAGGTGTCCAGTGTGAGG | |
| B6-F7 | CGCCGACATCATATGCNNNNNNNNCAGGTACAGCTGCAGCAGTCA | |
| B6-F8 | CGCCGACATCATATGCNNNNNNNNCAGGTGCAGCTGGTGGAGTCTGG | |
| B6-F9 | CGCCGACATCATATGCNNNNNNNNCAACTACAGGTGCCCACTCC | |
| B6-F10 | CGCCGACATCATATGCNNNNNNNNGAGGTGCAGCTGGTGGAGTCT | |
| Continued |  | |
| B1-IGM | AGCCGACAACCTTACGNNNNNNNNGCGGATGCACTCCCCTGARGAG | |
| B2-IGM | GTTCTGAGGTCATACGNNNNNNNNGCGGATGCACTCCCCTGARGAG | |
| B3-IGM | TCCAAACTCATCTACGNNNNNNNNGCGGATGCACTCCCCTGARGAG | |
| B4-IGM | GCAGGGATCTTATACGNNNNNNNNGCGGATGCACTCCCCTGARGAG | |
| B5-IGM | GTGGTATGGCTGTACGNNNNNNNNGCGGATGCACTCCCCTGARGAG | |
| B6-IGM | CTGCGTTCTGATTACGNNNNNNNNGCGGATGCACTCCCCTGARGAG | |
| P5 Primer-B1 | CGCCAAGTGTCTATGC | |
| P5 Primer-B2 | TGAGCGGATAACATGC | |
| P5 Primer-B3 | AAGCTGCGGAATATGC | |
| P5 Primer-B4 | ACGCCATATCGCATGC | |
| P5 Primer-B5 | TATAAGCAGAGCATGC | |
| P5 Primer-B6 | CGCCGACATCATATGC | |
| P3 Primer-B1 | AGCCGACAACCTTACG | |
| P3 Primer-B2 | GTTCTGAGGTCATACG | |
| P3 Primer-B3 | TCCAAACTCATCTACG | |
| P3 Primer-B4 | GCAGGGATCTTATACG | |
| P3 Primer-B5 | GTGGTATGGCTGTACG | |
| P3 Primer-B6 | CTGCGTTCTGATTACG | |
